# Virus-Host Interactions and Genetic Diversity of Antarctic Sea Ice Bacteriophages

**DOI:** 10.1128/mbio.00651-22

**Published:** 2022-05-09

**Authors:** Tatiana A. Demina, Anne-Mari Luhtanen, Simon Roux, Hanna M. Oksanen

**Affiliations:** a Molecular and Integrative Biosciences Research Programme, Faculty of Biological and Environmental Sciences, University of Helsinki, Helsinki, Finland; b Department of Microbiology, Faculty of Agriculture and Forestry, University of Helsinki, Helsinki, Finland; c Marine Research Centre, Finnish Environment Institute, Helsinki, Finland; d DOE Joint Genome Institute, Lawrence Berkeley National Laboratory, Berkeley, California, USA; e Helsinki Institute of Sustainability Science (HELSUS), Helsinki, Finland; Pacific Northwest National Laboratory

**Keywords:** Antarctic virus, infection cycle, metagenomics, sea ice, virus genome

## Abstract

Although we know the generally appreciated significant roles of microbes in sea ice and polar waters, detailed studies of virus-host systems from such environments have been so far limited by only a few available isolates. Here, we investigated infectivity under various conditions, infection cycles, and genetic diversity of the following Antarctic sea ice bacteriophages: *Paraglaciecola* Antarctic GD virus 1 (PANV1), *Paraglaciecola* Antarctic JLT virus 2 (PANV2), *Octadecabacter* Antarctic BD virus 1 (OANV1), and *Octadecabacter* Antarctic DB virus 2 (OANV2). The phages infect common sea ice bacteria belonging to the genera *Paraglaciecola* or *Octadecabacter*. Although the phages are marine and cold-active, replicating at 0°C to 5°C, they all survived temporal incubations at ≥30°C and remained infectious without any salts or supplemented only with magnesium, suggesting a robust virion assembly maintaining integrity under a wide range of conditions. Host recognition in the cold proved to be effective, and the release of progeny viruses occurred as a result of cell lysis. The analysis of viral genome sequences showed that nearly one-half of the gene products of each virus are unique, highlighting that sea ice harbors unexplored virus diversity. Based on predicted genes typical for tailed double-stranded DNA phages, we suggest placing the four studied viruses in the class *Caudoviricetes*. Searching against viral sequences from metagenomic assemblies, we revealed that related viruses are not restricted to Antarctica but are also found in distant marine environments.

## INTRODUCTION

Sea ice covers a significant area of polar oceans every year, affecting ocean ecology, biogeochemical cycles, and climate (1, 2). Sea ice, especially its liquid brines, is inhabited by various microorganisms, including viruses (3–7) that cope with temperatures below 0°C; rapidly changing salinity, pH, and nutrient concentrations; gas fluxes; and various light conditions (8). Metagenomic studies have revealed a high diversity and abundance of viruses in polar aquatic environments (9–11). Virus-like particle concentrations and virus-to-bacterium ratios in sea ice are typically higher than those in the surrounding seawater, suggesting active virus production in the ice (12–17). The role of viruses in controlling host abundance is significant in polar environments also due to the lower abundance and diversity of grazers (11, 18). Virus infections in sea ice are not restricted to lytic cycles but can include lysogenic ones (18, 19) and possibly pseudolysogeny (20). Viruses may also confer properties beneficial to their host survival (21). Low temperature environments in general are suggested to be hot spots of microbial evolution (20).

The studies of sea ice viruses have been limited typically to microscopic examinations and -omics approaches with only a few sea ice virus-host systems isolated, both from the Arctic and the Antarctic (21–24). All the known sea ice bacteriophage isolates display tailed icosahedral virions, except f327, which is filamentous (21–24). Among the sea ice tailed phages, all three types of tails have been observed, namely, long contractile, long noncontractile, and short noncontractile tails, which are characteristics of the myovirus, siphovirus, and podovirus morphotypes, respectively (22–24). Under laboratory conditions, the temperature range suitable for the growth of these virus-host systems varies, but the temperature at which the isolated sea ice phages are able to complete a productive infection cycle is typically lower than the maximal growth temperature for their host bacteria (22–24). The isolated sea ice viruses are host specific or have a narrow host range, and the known hosts are *Shewanella*, *Flavobacterium*, *Colwellia*, *Octadecabacter*, *Glaciecola*, and *Pseudoalteromonas* strains (21–24). The adsorption of two *Shewanella* phages was shown to be fast compared with that of mesophilic phages (25). The Baltic sea ice virus isolates have lytic infection cycles (25), while f327 does not lyse its *Pseudoalteromonas* host but affects its growth and physiological traits, which might be advantageous to host survival in the natural environment (21). Based on the genome comparisons, the six sequenced Baltic sea ice phage isolates are unrelated or distantly related to each other, except phages 1/4 and 1/40, which are also related to *Vibrio*-specific ICP1-like phages (25). In addition, putative proviral elements related to phage 1/44 were detected in the genomes of *Shewanella* sp. strains (25). The structural proteins of the Baltic sea ice phages recruited translated reads from metagenomic assemblies obtained from various aquatic environments and were not restricted to the Baltic Sea region (25). Taken together, the known sea ice phage isolate data suggest that sea ice environments harbor a diversity of phages with complex virus-host interactions, and they are related only distantly to phages from other environments.

Here, we studied four phages isolated from Antarctic sea ice (24) to understand their infectivity under various conditions, genetic diversity, and occurrence in different environments as well as to analyze their infection cycle parameters. A better understanding of the role of viruses in sea ice microbial communities would provide valuable information to be included in future sea ice biogeochemical models (26).

## RESULTS

### Antarctic sea ice phage isolates PANV1, PANV2, OANV1, and OANV2 tolerate elevated temperatures and lowered salinity.

**FIG 1:**
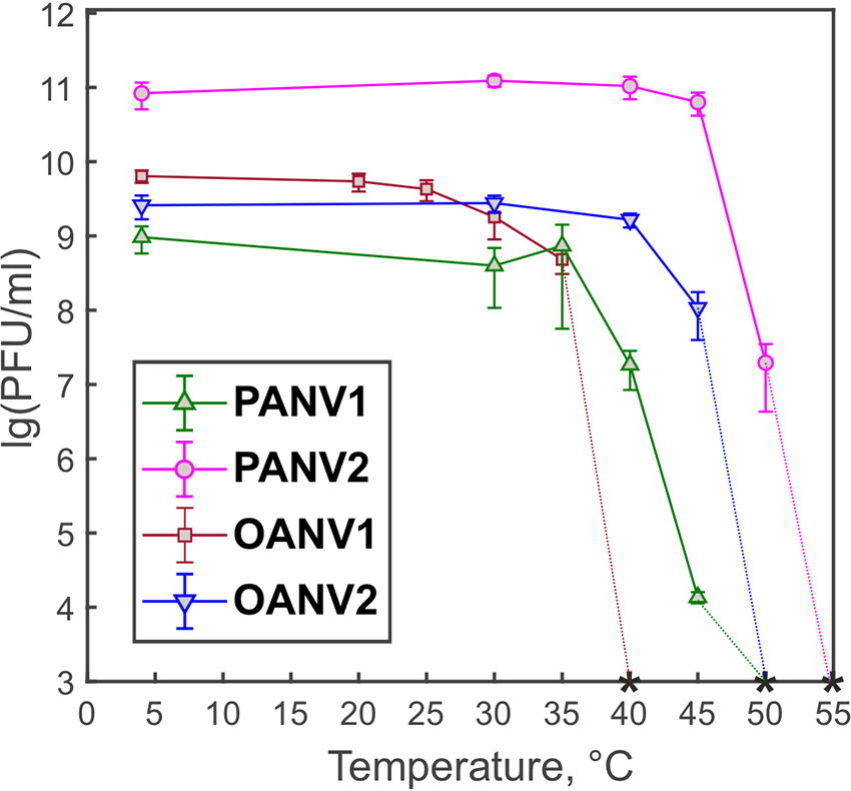
Virus infectivity after a 1-h exposure to different temperatures. Bars represent means of at least three independent replicates with standard error of the mean. Asterisks indicate that the titers are under the detection limit (<1 × 10^3^ PFU/mL).

To assess virus infectivity at different temperatures, viruses were incubated at 4°C to 55°C, using 4°C as a reference (100% infectivity) (Fig. 1). The studied viruses stayed fully infectious when exposed temporally to the temperatures up to 30°C or even higher. No statistically significant difference was observed in PANV1 infectivity at 4°C and 35°C, whereas the titer dropped to ~2% at 40°C and only ~0.001% of particles were infective at 45°C. For PANV2, the 45°C temperature had no statistically significant effect on the infectivity, but a sharp titer drop to ~0.03% was observed at 50°C. OANV1 preserved the titer at 25°C, while at 30°C and 35°C, the infectivity was ~28% and ~7.5%, respectively. For OANV2, no statistically significant difference in titers at 4°C and 40°C was observed, but only ~4% of particles were infective after incubating at 45°C. Virus titers decreased 1/10th or more at 40°C, 50°C, 35°C, and 45°C for PANV1, PANV2, OANV1, and OANV2, respectively. The titers were under the detection limit (<1 × 10^3^ PFU/mL), at 50°C, 55°C, 40°C, and 50°C for PANV1, PANV2, OANV1, and OANV2, respectively (Fig. 1).

**FIG 2:**
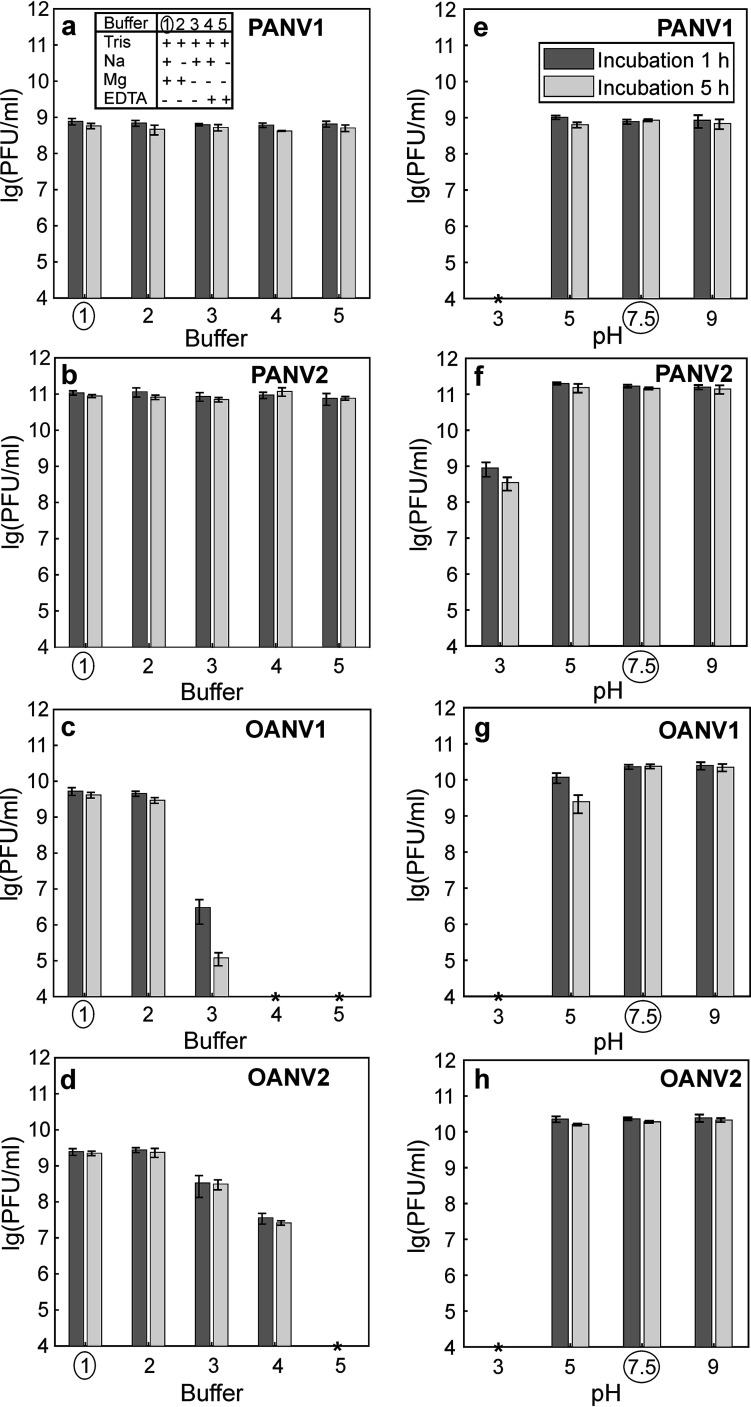
Virus infectivity in buffers with different ion composition and pH. Virus stocks were diluted either in SM buffer (circle in all panels), modified SM buffers where some components were omitted (key in a) (a to d), or modified SM buffers of different pHs (e to h). The incubation lasted 1 h (dark gray) and 5 h (light gray). Bars represent means of at least three independent replicates with standard error of the mean. An asterisk indicates that the titer is under the detection limit (<1 × 10^4^ PFU/mL).

All four viruses studied here remained infectious without NaCl (Fig. 2a to d, buffer 2). Moreover, PANV1 and PANV2 infectivities were preserved in the absence of both NaCl and Mg^2+^, even if residual Mg^2+^ ions were removed by the chelating agent ethylenediaminetetraacetic acid (EDTA) (Fig. 2a and b, buffers 3 to 5). When only MgSO_4_ was excluded from the saline-magnesium (SM) buffer, the OANV1 titer dropped by 4 to 5 orders of magnitude, and the OANV2 titer dropped to 1/10th (Fig. 2c and d, buffer 3). If all residual Mg^2+^ ions were removed using EDTA (Fig. 2c and d, buffer 4), the OANV1 titer was under the detection limit (<1 × 10^4^ PFU/mL) and the OANV2 titer dropped 100-fold. When both NaCl and MgSO_4_ were removed (Fig. 2c and d, buffer 5), OANV1 and OANV2 titers were <1 × 10^4^ PFU/mL.

No statistically significant difference in virus titers was observed when virus stocks were incubated in SM buffer (pH 7.5) with either Tris-HCl or NaH_2_PO_4_ (see Fig. S1 in the supplemental material). For all four viruses, pH 9 had no significant effect on the infectivity (Fig. 2e to h). PANV1, PANV2, and OANV2 were also stable at pH 5. OANV1 preserved the titer at pH 5 after 1 h of incubation, but the titer dropped 10-fold after 5 h. At pH 3, the titers dropped significantly for all four viruses (PANV2, <1%; others, <1 × 10^4^ PFU/mL).

10.1128/mbio.00651-22.6 FIG S1 Stability of virus infectivity in SM buffer containing NaH_2_PO_4_ (buffer 1) or Tris-HCl (buffer 2; pH 7.5). Virus stocks were diluted 1,000-fold in the buffers and incubated 1 h (dark gray) and 5 h (light gray). Bars represent means of at least three independent replicates with standard error of the mean shown as error bars. Download FIG S1, PDF file, 0.1 MB. Copyright © 2022 Demina et al. 2022 Demina et al. https://creativecommons.org/licenses/by/4.0/ This content is distributed under the terms of the Creative Commons Attribution 4.0 International license.

### Antarctic sea ice phages PANV1, PANV2, OANV1, and OANV2 adsorb effectively to their hosts.

**FIG 3:**
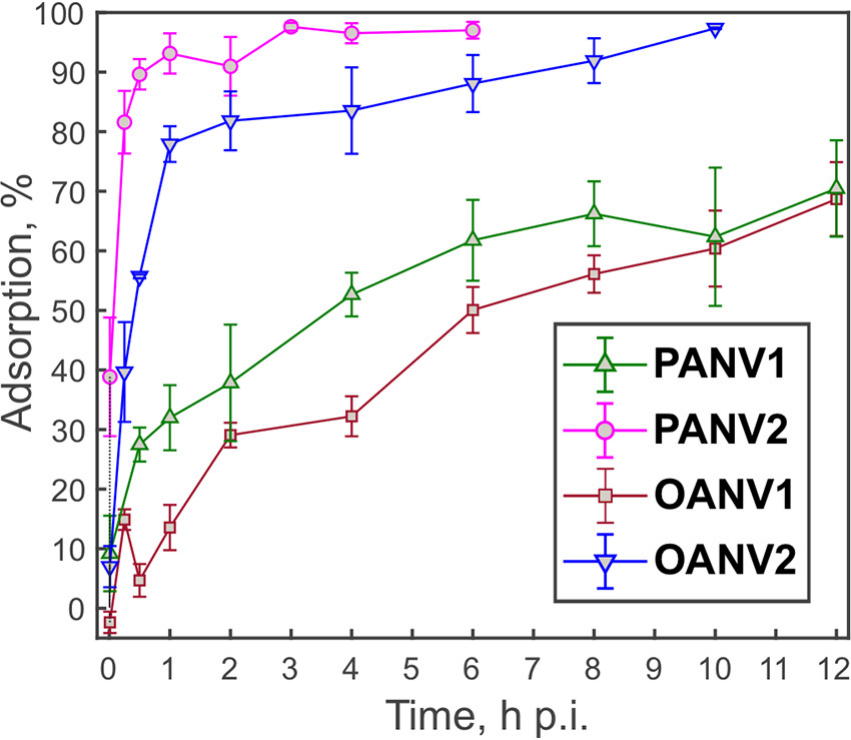
Adsorption efficiency shown as a percentage of bound viruses at 4°C. Means of at least three independent replicates are presented with standard error of the mean.

PANV1, PANV2, OANV1, and OANV2 adsorbed effectively to their hosts, achieving at least ~50% binding efficiency within 6 h at 4°C (Fig. 3). During the experimental set-up, the viruses were not inactivated since no decrease in virus plaque numbers was observed in virus control samples. PANV2 and OANV2 showed the fastest and the most efficient adsorption, having ~80% particles adsorbed by 30 min and 1 h postinfection (p.i.), respectively, and reaching ~100% binding later (Fig. 3). The adsorption rate constant *k* calculated for the first 30 min p.i. (*n* = 3) was 3.9 × 10^−9^ and 9.0 × 10^−12 ^mL/min for PANV2 and OANV2, respectively. PANV1 and OANV1 adsorbed with the rates of 5.4 × 10^−10^ and 4.6 × 10^−13 ^mL/min, respectively, reaching ~70% adsorption by 12 h p.i.

### Infection cycles of PANV1, PANV2, OANV1, and OANV2 phages result in cell lysis.

**FIG 4:**
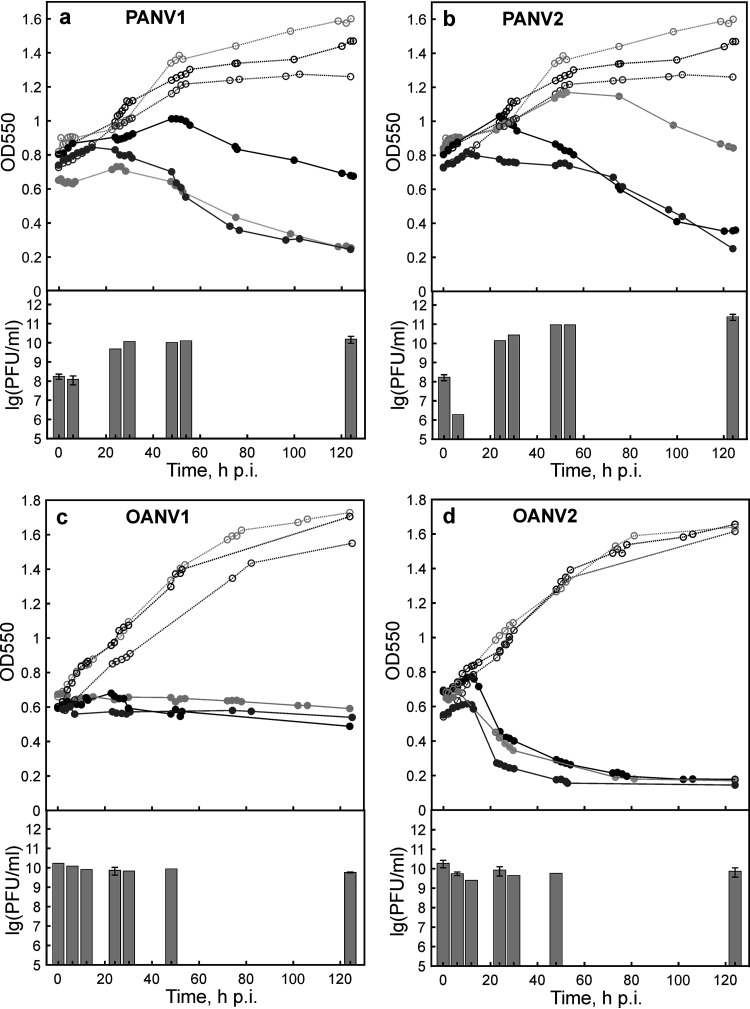
One-step growth curves of PANV1 (a) and PANV2 (b) in *Paraglaciecola* IceBac 372, OANV1 in *Octadecabacter* IceBac 419 (c), and OANV2 in *Octadecabacter* IceBac 430 (d). Growth curves of uninfected (open circles, dashed lines) and infected (closed circles, solid lines) cultures from three independent repeats are shown at the top graphs. Curves with same shades of gray represent the same repeat. The numbers of free viruses are shown as bars in the bottom graphs, with means with standard error of the mean where appropriate (*n* = 3) or otherwise means of *n* = 2.

Bacterial strains *Paraglaciecola* IceBac 372 and *Octadecabacter* IceBac 419 and 430 grew to early stationary stage in 4 to 7 days (from an optical density of 550 nm [OD_550_] of 0.2 to 1.4 to 1.6), showing typical growth curves of a bacterial culture. The cultures were infected at the logarithmic growth phase (IceBac 372, OD_550_ of 0.8, ~2 × 10^7^ CFU/mL; IceBac 419 and IceBac 430, OD_550_ of 0.6, ~3 × 10^9^ CFU/mL) using a multiplicity of infection (MOI) of 8 to 10 to analyze the one-step growth of the phages (Fig. 4). Uninfected cultures reached an OD_550_ of 1.6 to 1.7 by 124 h p.i. (Fig. 4). The turbidities of cultures infected with PANV1, PANV2, or OANV2 started to decrease at 12 to 20 h p.i. and eventually dropped to 0.2 to 0.4, indicating cell lysis (Fig. 4a, b, and d). The optical density of the OANV1-infected culture stayed at the same level as that at the time of infection (Fig. 4c). For PANV1 and PANV2, an increase in the numbers of free viruses was detected at 24 h p.i., suggesting progeny virus production. The lysate titers were ~1.6 × 10^10^ and ~2.4 × 10^11^ PFU/mL for PANV1 and PANV2, respectively. In OANV1 and OANV2 infections, the increase of free viruses could not be detected with the methods used here. However, for OANV1, the number of viable *Octadecabacter* IceBac 419 cells at the time of infection (~1.1 × 10^9^ CFU/mL) reduced almost 2 orders of magnitude as a result of the virus addition (~2.3 × 10^7^ CFU/mL) by 124 h p.i., while the number of viable cells in the uninfected culture of IceBac 419 was growing (~3.3 × 10^9^ CFU/mL by 124 h). This result can be interpreted as lysis being caused by the virus infection. Similarly, the numbers of viable cells in the OANV2-infected IceBac 430 culture dropped noticeably (from ~1.4 × 10^9^ at 0 h p.i. to ~5 × 10^6^ CFU/mL at 124 h p.i.) compared with those of the uninfected culture (~3.5 × 10^9^ CFU/mL at 124 h p.i.).

### The four Antarctic sea ice phage isolates have largely unique genomes.

**FIG 5:**
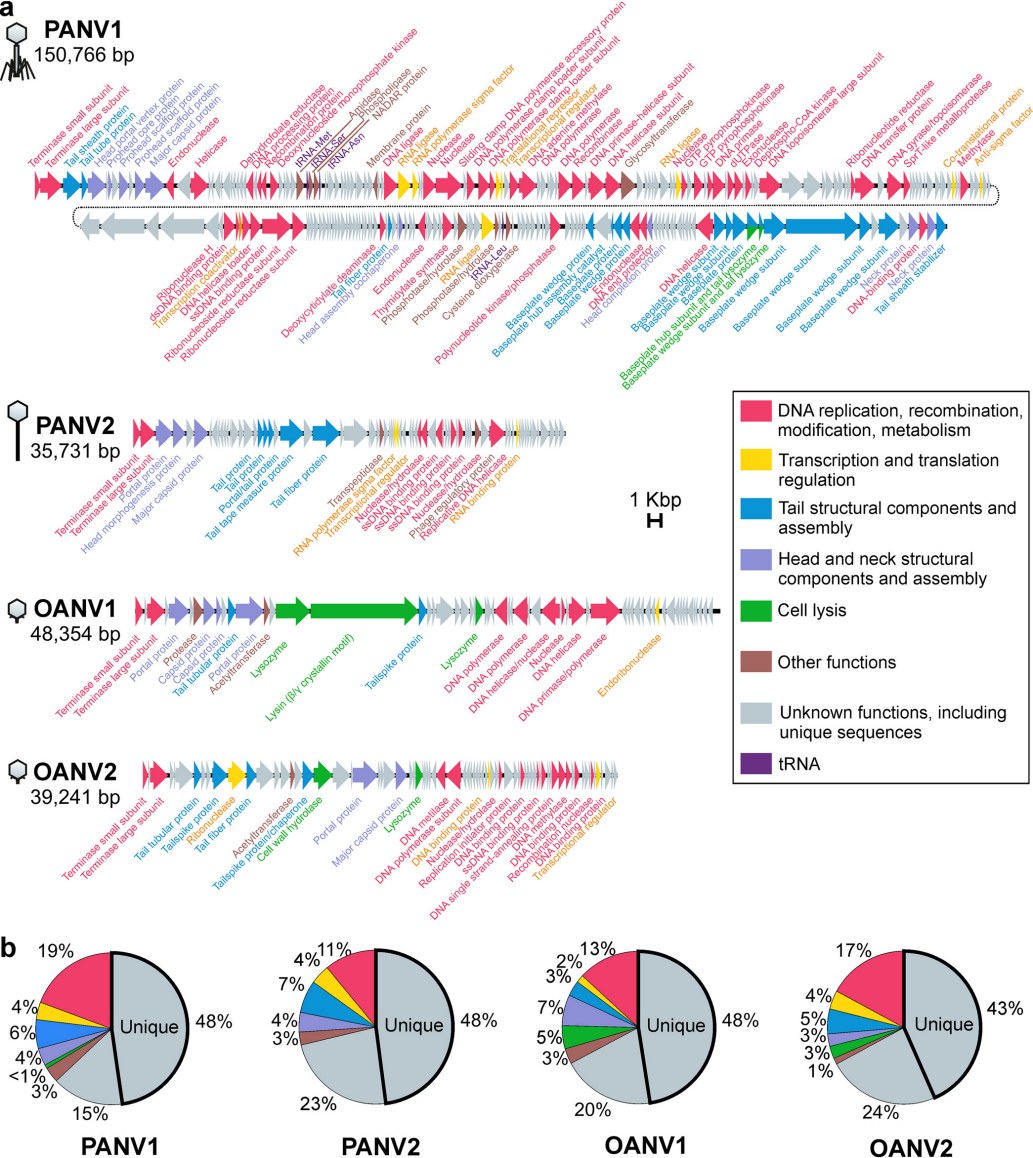
(a) Genomes of PANV1, PANV2, OANV1, and OANV2. ORF colors refer to the assigned functional categories (inset, same for a and b). Virus morphotypes are indicated schematically on the left. (b) Distribution of functional categories assigned to the protein-coding ORFs of PANV1, PANV2, OANV1, and OANV2. The portions of unique ORF products not having homologs in the NCBI nr protein database are outlined with bold.

PANV1, PANV2, OANV1, and OANV2 genomes are double-stranded DNA (dsDNA) molecules ranging from ~36 to ~151 kb, with GC content of 38% to 62% and 61 to 243 predicted protein-coding open reading frames (ORFs) (Table 1, Fig. 5a; see Tables S1 to S4 in the supplemental material). Of the phages studied here, only PANV1 contains predicted tRNA genes (Table 1). All four virus genomes include ORFs encoding small and large terminase subunits, which are hallmark genes for tailed dsDNA bacteriophages that package their genomes into a preformed procapsid (27). ORF numbering in the four genome sequences studied here was started with the ORF for the small terminase subunit (ORF1).

**TABLE 1:** Antarctic sea ice viruses used in this study

Virus^*a*^	Virus morphotype (head diam [nm])	Host strain^*a*^	Virus genome information^*b*^				
			Length (bp)	GC content (%)	No. of ORFs	No. of tRNAs	GenBank accession no
*Paraglaciecola* Antarctic GD virus 1 (PANV1)	Icosahedral head, long contractile tail, (myovirus type), 71 ± 7^*a*^	*Paraglaciecola* IceBac 372	150,766	37.7	243	4	MW805361
*Paraglaciecola* Antarctic JLT virus 2 (PANV2)	Icosahedral head, long noncontractile tail (siphovirus type), 52 ± 8^*a*^	*Paraglaciecola* IceBac 372	35,731	41.0	73	0	MW805362
*Octadecabacter* Antarctic BD virus 1 (OANV1)	Icosahedral head, short noncontractile tail (podovirus type), 68^*b*^	*Octadecabacter* IceBac 419	48,354	62.1	61	0	MW805363
*Octadecabacter* Antarctic DB virus 2 (OANV2)	Icosahedral head, short noncontractile tail (podovirus type), 53 ± 7^*a*^	*Octadecabacter* IceBac 430	39,241	53.3	76	0	MW805364

a Data are from reference 24.

b Data are from this study.

10.1128/mbio.00651-22.1 TABLE S1 Putative functions assigned to PANV1 ORF products. Download Table S1, PDF file, 0.3 MB. Copyright © 2022 Demina et al. 2022 Demina et al. https://creativecommons.org/licenses/by/4.0/ This content is distributed under the terms of the Creative Commons Attribution 4.0 International license.

The majority of predicted ORF products (63% to 71%) of the four virus genomes could not be assigned with any functions, including unique sequences (43% to 48%) that had no homologues in the NCBI nonredundant (nr) protein database (Fig. 5b). Surprisingly, the ratio between unique ORF products and those that had some homologous sequences in the database was about the same for all four viruses, regardless of the genome length (Fig. 5b).

About 29% to 37% of the studied sequences had homologs in the database, allowing us to predict their functions. BLAST matches revealed mosaic similarities to the sequences of other phages, as well as moderate and psychrophilic bacteria. The following functional categories were assigned to the gene products (gps): (i) proteins involved in DNA replication, recombination, modification, and metabolism; (ii) transcription and translation regulation proteins; (iii) virion and tail structural components; (iv) cell lysis; and (v) other functions (Tables S1 to S4; Fig. 5). Some putative proteins could be classified to more than one category.

PANV2 gp55 has significant blast hits to phage regulatory Rha proteins, encoded by temperate phages (28, 29). Unlike the other three viruses, no lysis genes were predicted in PANV2. In OANV1, gp15 and gp16 are putatively lysis-related proteins. Noticeably, gp16 is the longest predicted OANV1 protein (2,986 residues), and it is similar to *Bordetella* phage BPP-1 bbp10, which is a lysin containing a beta/gamma crystalline motif (blastp search 19.02.21, 50% cover, 30% identity, E value of 3e-128). In PANV1, gp232 and gp233 were annotated as baseplate subunits having lysozyme activity, being similar to the gene products in T4-like phages (T4 gp5 and gp25, respectively) (30, 31). In OANV2, gp19 is presumably a cell wall hydrolyzer and gp29 is a lysozyme.

Along with the categories typical for dsDNA phage genomes, additional functions were also predicted. PANV1 gp36 was predicted to be a phospholipase (HHpred search, hit 1LWB_A, probability 98.8, E value of 2e-8, 19.2.2021) and thus is possibly involved in lipid metabolism. PANV1 gp37 putatively belongs to the NAD and ADP-ribose (NADAR) superfamily, having a hydrolyzer activity and taking part in carbohydrate derivative metabolic processes (HHpred search, hit 2B3W_A, probability 100, E value of 7.1e-34, 19.02.2021). PANV1 gp51 had matches to mechanosensitive channel proteins, which are involved in transmembrane transport (e.g., HHpred hit to 6RLD_D, probability 99.7, E value of 7.2e-17, 19.02.2021). However, a transmembrane helix was predicted in this protein with only ~0.6 posterior probability by TMHMM v. 2.0. In PANV2, gp30 is a putative transpeptidase, which is involved in peptidoglycan cross-linking (HHpred, 4LPQ_A, probability 96.6, E value of 0.01, 19.02.2021).

Based on the Virfam analysis of the neck module and part of the head and tail proteins (32), PANV1 was assigned to the category of “*Myoviridae* of Type 2,” adopting the structural organization of the myophage T4 neck. PANV2 was assigned to “*Siphoviridae* of Type 1 cluster 5,” adopting the structural organization of the siphophage SPP1 neck. Both OANV1 and OANV2 were predicted to belong to “*Podoviridae* of Type 3,” adopting the structural organization of the podophage P22 neck. (32). The Virfam-based classification is consistent with the tail morphology determined by transmission electron microscopy previously for PANV1, PANV2, and OANV2 (24) and here for OANV1 (see below).

### OANV1 is a podovirus.

Since the sequence information indicated that OANV1 is not a siphovirus, we reanalyzed the OANV1 virus morphology. Transmission electron micrographs of purified OANV1 particles displayed a podovirus-like morphotype, as follows: tailed virions with icosahedral heads (diameter, ~68 nm; *n* = 44) and short noncontractile tails (length, ~11 nm; *n* = 25) (see Fig. S2a and b in the supplemental material). Electron micrographs were taken from two independently purified virus samples (specific infectivity, 6.1 × 10^13^ and 3.7 × 10^13^ PFU/mg of protein) using two negative stains and all demonstrated consistent particle morphology. The protein patterns of the purified OANV1 particle samples were identical to each other (Fig. S2c) and similar to that reported previously (24). OANV1 was identified initially as a siphovirus, most probably due to some error during the sample preparation and imaging (24).

10.1128/mbio.00651-22.7 FIG S2 Transmission electron micrographs of OANV1 virus particles stained with uranyl acetate (2% [w/v]) (a) or Nano-W (b). Scale bar is 100 nm in a for a and b. (c) Polyacrylamide gel electrophoresis of purified virus samples which were prepared in parallel (labeled 1 and 2; 10 μg each) and used in transmission electron microscopy in a and b, respectively. M, marker (PageRuler unstained protein ladder). Download FIG S2, PDF file, 0.1 MB. Copyright © 2022 Demina et al. 2022 Demina et al. https://creativecommons.org/licenses/by/4.0/ This content is distributed under the terms of the Creative Commons Attribution 4.0 International license.

### Viruses related to the known Antarctic sea ice phage isolates are found in Antarctica and in other distant marine environments.

The overall nucleotide identity between four genomes is the lowest (~24%) between PANV1 and PANV2 and the highest (~52%) between OANV1 and OANV2. When the complete sequences were used as queries in blastn searches (somewhat similar sequences option, dated 19 February 2021) against all virus sequences (NCBI taxonomy identifier [ID] 10239) in the nr nucleotide collection, typically only less than 3% of the whole-genome sequences could be aligned with other virus sequences available, highlighting the overall uniqueness of these Antarctic sea ice virus isolates. One exception was the match of the OANV2 genome to the uncultured *Caudovirales* phage genome assembly (GenBank accession number LR798304), which was obtained from metagenomes from the Římov Reservoir (freshwater human-made pond), Czech Republic (33). The overall identity between the genomes was 52% (see Fig. S3a in the supplemental material).

10.1128/mbio.00651-22.8 FIG S3 OANV2 and the selection of similar virus genome sequences, as follows: uncultured *Caudovirales* phage (GenBank accession number LR798304.1) found with blastx search against nr protein database (a) and scaffolds found with blastn search against IMG/VR database (b). A full list of scaffolds is presented in Table S5. Here, those scaffolds that were identical to a part of some other scaffold are excluded. ORFs and genes are shown as arrows, and regions that are similar between sequences are shown as shadings; blastn, E value threshold of 0.001, gray for direct and red for inverted similarities, from 63% to 100% (a) or 69% to 100% (b). Note that OANV2 and some other sequences are reversed in b. Color codes for OANV2 ORFs are shown in the bottom. Sampling locations are marked on the right. The figure was generated using Easyfig v. 2.2.2. Download FIG S3, PDF file, 1.5 MB. Copyright © 2022 Demina et al. 2022 Demina et al. https://creativecommons.org/licenses/by/4.0/ This content is distributed under the terms of the Creative Commons Attribution 4.0 International license.

10.1128/mbio.00651-22.2 TABLE S2 Putative functions assigned to PANV2 ORF products. Download Table S2, PDF file, 0.1 MB. Copyright © 2022 Demina et al. 2022 Demina et al. https://creativecommons.org/licenses/by/4.0/ This content is distributed under the terms of the Creative Commons Attribution 4.0 International license.

10.1128/mbio.00651-22.3 TABLE S3 Putative functions assigned to OANV1 ORF products. Download Table S3, PDF file, 0.2 MB. Copyright © 2022 Demina et al. 2022 Demina et al. https://creativecommons.org/licenses/by/4.0/ This content is distributed under the terms of the Creative Commons Attribution 4.0 International license.

10.1128/mbio.00651-22.4 TABLE S4 Putative functions assigned to OANV2 ORF products. Download Table S4, PDF file, 0.1 MB. Copyright © 2022 Demina et al. 2022 Demina et al. https://creativecommons.org/licenses/by/4.0/ This content is distributed under the terms of the Creative Commons Attribution 4.0 International license.

10.1128/mbio.00651-22.5 TABLE S5 Scaffolds with several (at least three) regions recruited as hits in the blast search against IMG/VR with Antarctic virus isolates whole genomes as queries (dated 9 December 2020). Download Table S5, PDF file, 0.1 MB. Copyright © 2022 Demina et al. 2022 Demina et al. https://creativecommons.org/licenses/by/4.0/ This content is distributed under the terms of the Creative Commons Attribution 4.0 International license.

**FIG 6:**
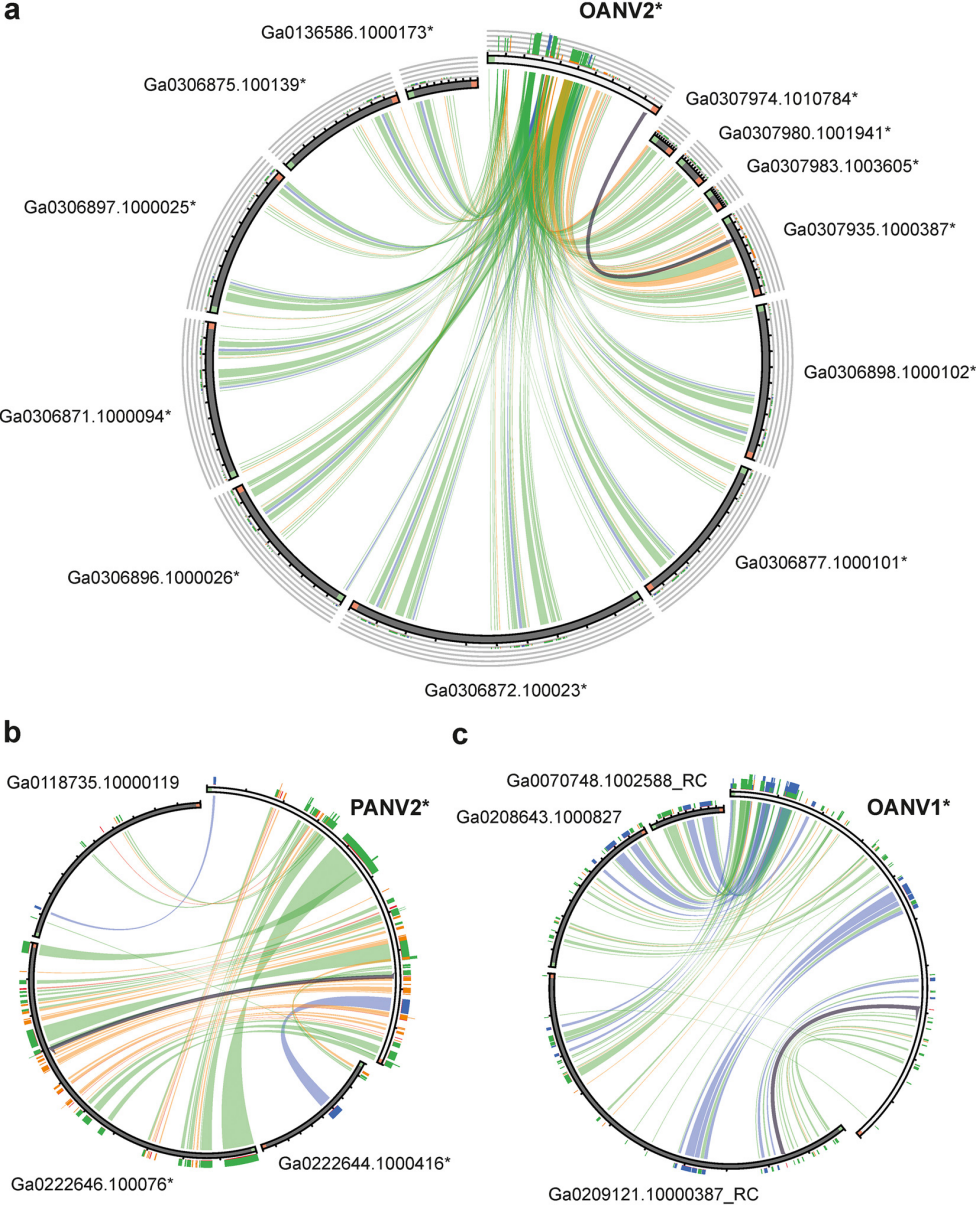
Visualizing sequence similarities between OANV2 (a), PANV2 (b), and OANV1 (c) and corresponding metagenome-derived scaffolds found in IMG/VR database (see Table S5). The figure was generated with Circoletto, coloring ribbons by % identity with absolute coloring as follows: blue, ≤80; green, ≤90; orange, ≤95; and red, >95. Minimal and maximal identity intervals are 77.9% to 100.0% (a), 79.2% to 100.0% (b), and 78.3% to 97.0% (c). The orientation of the sequences is clockwise; in addition, the sequence starts are marked with green and ends with red. Some sequences are presented as reverse complements (RC). Sequences originating from Antarctica are marked with an asterisk.

In contrast, a similar blastn search with PANV1, PANV2, OANV1, and OANV2 whole-genome sequences as queries against the Integrated Microbial Genomes/Virus (IMG/VR) (34) database resulted in many hits to sequences obtained from various locations and environments. We have given a priority to the search based on the whole-genome sequences rather than separate ORFs to ensure more specific hits and the possibility to easily select matches with several regions of similarity. Both regions containing ORFs with assigned functions and unknown ones recruited hits. Most blastn hits covered relatively short regions (38 to 3,870 nucleotides [nt]); hence, to find similar viral genomes rather than separate ORFs, scaffolds with at least three regions of similarity were selected for further analysis (see Table S5 in the supplemental material). While PANV1 had no such related scaffolds, the other three virus genome sequences recruited several scaffolds originating from Antarctica and other environments (Fig. 6). Notably, some of the selected scaffolds represented identical parts of each other and originated from the same project and sampling location and so likely represented sequencing of the same virus across multiple samples (the duplicates excluded in Fig. 6).

PANV2 was similar to scaffolds assembled from saline water microbial communities from Ace Lake, Antarctica (35), and one scaffold from marine sediment microbial communities from methane seeps sampled in Hudson Canyon, US Atlantic Margin. OANV1 was related to scaffolds from oil-polluted marine microbial communities from Coal Oil Point, Santa Barbara, CA (36, 37), and aqueous microbial communities from the Delaware River and Bay (38), but no samples from Antarctica (Fig. 6). On the contrary, OANV2 was similar only to scaffolds originating from saline lake microbial communities from the following various locations in Antarctica: Organic Lake, Club Lake, Deep Lake, and Saline Lake on Rauer Islands (35, 39, 40). Similarity regions are distributed along the whole virus genomes and include ORFs assigned with different functions, including those encoding capsid structural proteins (see Fig. S3b, S4, and S5 in the supplemental material). The overall nucleotide identity between the query genomes and scaffolds retrieved from the database was ~40% for those sequences that have a similar length (Table S5).

10.1128/mbio.00651-22.9 FIG S4 PANV2 and the selection of similar scaffolds found with blastn search against IMG/VR database. A full list of scaffolds is presented in Table S5. Here, those scaffolds that were identical to a part of some other scaffold are excluded. ORFs and genes are shown as arrows, and regions that are similar between sequences are shown as shadings (blastn, E value threshold of 0.001, gray for direct and red for inverted similarities, from 66% to 100%). Note that the PANV2 genome is shown rearranged starting with nucleotide 17375. Color codes for PANV2 ORFs are shown in the bottom. Sampling locations are marked on the right. The figure was generated using Easyfig v. 2.2.2. Download FIG S4, PDF file, 0.2 MB. Copyright © 2022 Demina et al. 2022 Demina et al. https://creativecommons.org/licenses/by/4.0/ This content is distributed under the terms of the Creative Commons Attribution 4.0 International license.

10.1128/mbio.00651-22.10 FIG S5 OANV1 and the selection of similar scaffolds found with blastn search against IMG/VR database. A full list of scaffolds is presented in Table S5. Here, those scaffolds that were identical to a part of some other scaffold are excluded. ORFs and genes are shown as arrows, and regions that are similar between sequences are shown as shadings (blastn, E value threshold of 0.001, gray for direct and red for inverted similarities, from 65% to 100%). Color codes for OANV1 ORFs are shown in the bottom. Sampling locations are marked on the right. The figure was generated using Easyfig v. 2.2.2. Download FIG S5, PDF file, 0.1 MB. Copyright © 2022 Demina et al. 2022 Demina et al. https://creativecommons.org/licenses/by/4.0/ This content is distributed under the terms of the Creative Commons Attribution 4.0 International license.

A blast search of PANV1, PANV2, OANV1, and OANV2 genome sequences against the IMG/VR spacer databases did not yield any information about potential additional hosts of these viruses. No reliable hits were detected to CRISPR spacers from isolate genome, and the hits to metagenome-derived spacers were on short metagenome contigs that could not be affiliated taxonomically.

## DISCUSSION

### The effect of elevated temperature, low ionic strength, and acidic/alkaline conditions on viral infectivity of the known sea ice phage isolates.

The sea ice phages studied here were able to cope with temporal exposures to the temperatures of at least 30°C or even 50°C (Fig. 1), which is much higher than their growth temperature (0°C to 5°C) (24). A similar physical stability of virions has been observed in the tailed dsDNA phages isolated from the Baltic sea ice on *Shewanella* or *Flavobacterium* sp. (23, 24) and cold-active phages isolated from Napahai Wetland and Mingyong Glacier, China ([Bibr B41][Bibr B42][Bibr B45]). In comparison, the cold-active tailed dsDNA phage 9A of Colwellia psychrerythraea from the Arctic nepheloid layer is inactivated rapidly at 25°C to 33°C with no effect of salinity or clay particles on thermal lability (46). The sea ice viruses studied here demonstrated no infectivity losses in the absence of NaCl, which is the main salt in marine water. Moreover, PANV1 and PANV2 infectivity was not affected by the absence of Mg ions either. Maintaining infectivity in the absence of salts as well as in a wide pH range may be beneficial for viral survival in sea ice brine channels, where salinity and pH may change across the brine network (6, 8). Some bacteriophages isolated from solar salterns, where salinity may change considerably following evaporation and rainfall events, also have wide salinity tolerance ranges (47, 48), while others are more sensitive to lowered salinity (49). Similar to the observed lack of typical patterns in virion stability of cold-active phages, different patterns in the variation of infectivity have been observed in bacteriophages in general and do not depend on virus morphotype or taxonomic assignments (50). Virus thermal inactivation may be caused by the release of genetic material from the capsid, as well as DNA and protein denaturation (51). Bacteriophages may aggregate at pH levels lower than their isoelectric point (52) or in lowered ionic strength conditions (53). The stability of PANV2 under different conditions shown here makes it an attractive model for future studies of molecular adaptation and virion architecture of viruses residing in sea ice.

### Host interactions of the Antarctic sea ice virus isolates.

Viruses isolated from Arctic, Baltic, and Antarctic sea ice so far seem to be very host specific and have different temperature limits for successful host infection, as tested under laboratory conditions (22–24). No new potential hosts could be assigned to the viruses studied here using the IMG/VR spacers databases, which likely reflects the specificity of sea ice phages and the limited representation of Antarctic microbes in the current genome and CRISPR spacer data. The relatively fast adsorption of PANV2 (90% in 30 min) and slower adsorption of PANV1 (maximally ~70% in 12 h) resulted in nonsynchronized lysis and active virus production in *Paraglaciecola* IceBac 372 observed at 24 h p.i. Putative tail fiber proteins identified in PANV1 may have roles in the attachment to host cells. The similarity of the PANV1 gp232 and gp233 to lysozymes in T4-like phages suggests similar lysis mechanisms (30, 31), whereas siphovirus PANV2 may use some unknown lysis mechanisms since no hits to known lysis-related proteins were identified or its efficient lysis might be dependent on another resident phage (54). Lysogeny seems to be prevalent in polar regions (19, 55, 56). The similarity of PANV2 gp55 to Rha regulatory proteins found in lambdoid phages and bacterial prophage regions (28, 29) suggests that PANV2 may establish a lysogenic infection cycle. Switches between infection modes can be triggered by environmental factors as well as the physiology of host cells (57).

For *Octadecabacter* podoviruses, OANV2 adsorption was faster and more efficient than that of OANV1 to their respective host strains. Putative tail spike proteins, possibly involved in the attachment to host cells, were found both in OANV1 and OANV2. OANV2 infection resulted in a sharp cell density decrease at 12 h p.i., while no decrease in the optical density was observed for OANV1-infected culture. Nonetheless, the number of viable cells decreased during both infections, suggesting cell lysis, which is also supported by the presence of putative lysis-related genes in OANV1 and OANV2 genomes.

Adsorption rate constants of four Antarctic sea ice viruses varied noticeably from the fast binder PANV2 to the slow binder OANV1 with the PANV1 adsorption rate of 5.4 × 10^−10 ^mL/min being the most similar to that observed previously in *Shewanella* phages isolated from Baltic sea ice, namely, phages 1/4 and 3/49, at 4°C (25). Infection cycles of the phages 1/4 and 3/49 were, however, considerably faster (measured at 15°C) (25). Cold-active tailed bacteriophages from Napahai Wetland and Mingyong Glacier, China, have optimal plaque formation at 15°C to 20°C, rapid and efficient adsorption, and short latent period, as well as fast and complete lysis (41–45). Thus, Antarctic sea ice phages studied here have effective but relatively slow infections, which may be due to slow host growth rates and preferred low temperatures (24). Similarly, slow infections with long latent periods have been observed for the *Pseudoalteromonas* phage from the North Water, Arctic, (15 h at 0°C) (58) and Pseudomonas putrefaciens phage 27 from Boston harbor water (8.5 and 14 h at 2°C on strains P10 and P19X, respectively) (59). In the case of cold-active bacteriophage 9A infection in Colwellia psychrerythraea 34H, the latent period was shown to differ depending on the growth temperatures, ranging from a few hours at 8°C to several days at temperatures below zero (60). Phage adsorption rates and latent period length may also depend on the host preincubation temperature (59, 61).

### Genetic diversity of Antarctic sea ice virus isolates and possible links to other biomes.

Viral diversity seems to be unique for Antarctica but is generally lower than that in lower-latitude marine systems (62). Overall, Antarctic phages studied here are genetically diverse and not closely related one to another or to any other sequenced sea ice phage isolates (22, 25, 63). The detected genetic similarities are rather mosaic and are not restricted to cold-active microorganisms. The identified functional categories are typical for tailed dsDNA phages, and we suggest placing the four studied viruses in the class *Caudoviricetes*. A few PANV1 proteins may take part in lipid and carbohydrate metabolism and membrane transport, thus possibly being auxiliary metabolic genes, which is seen commonly in marine phages (64). A high percentage of unique sequences in the Antarctic sea ice virus isolates emphasizes that the genetic diversity of sea ice viruses remains largely unexplored. Similarly, ocean viromes contain a high number of sequences having no homologs in reference databases (65).

Only a few sea ice virus isolates with sequenced genomes are available, and additionally, metagenomics-based studies addressing viral diversity in the Southern Ocean are also still scarce (19, 62). Exploring metagenome-derived viral sequences deposited in the IMG/VR database with four Antarctic sea ice virus genomes as queries showed that similar sequences may be found across different geographically distant environments. Obviously, ice melting may increase the transmission of viruses from Antarctica, e.g., from ancient glacial lakes to sea ice and seawater. It is, thus, intriguing to see whether future samplings performed in Antarctica and beyond would shed light on the global distribution of viruses related to the Antarctic sea ice phages studied here.

## MATERIALS AND METHODS

### Growth conditions and virus infectivity.

Viruses and bacteria (Table 1) were grown aerobically at 4°C or 5°C in Zobell Reef crystal (RC) medium as described previously (24). The effect of temperature on virus infectivity was tested by incubating virus stocks at 4°C to 55°C for 1 h. The effects of Na^+^ and Mg^2+^ ions were assessed by diluting virus stocks 1,000-fold in SM buffer (50 mM Tris-HCl [pH 7.5], 100 mM NaCl, and 8 mM MgSO_4_) (22), SM buffer lacking either NaCl or MgSO_4_, or both, or lacking MgSO_4_ but supplemented with 10 mM EDTA and incubating at 4°C for 1 and 5 h. The effect of pH was tested similarly using SM buffer either with 50 mM NaH_2_PO_4_ (pH 3, 5, and 7.5) or Tris-HCl (pH 7.5 and 9). After all incubations, virus infectivity was assessed by plaque assay as described previously (24). A single-factor analysis of variance (ANOVA) test was used when three or more groups were compared or a *t* test (two-sample assuming equal variances) when two groups were compared. Groups were considered statistically not different if the *P* value was >0.05.

### Adsorption and infection cycle.

To determine adsorption efficiency and rates, exponentially growing host cultures (OD_550_ of ~0.8) were infected with a multiplicity of infection (MOI) of ~0.001 and incubated aerobically at 4°C. Samples in which cells were replaced with broth were used as controls. To determine the number of unbound viruses, samples were diluted in 4°C broth (1:10 or 1:100), cells were removed (Eppendorf table centrifuge, 16,200 × *g*, 5 min, and 4°C), and supernatants were subjected to plaque assay. The percentage of adsorption was calculated from all (particles in broth) and unbound particles (in infected cultures) as follows: % bound particles = [(all – unbound)/all] × 100%. Adsorption rate constant was calculated as (*k*) = [2.3/(*B* × *t*)] × log(*p*0/*p*), where *B* is cell concentration, *p*0 and *p* are free virus concentrations at time point zero and after time period *t*, respectively (66).

For life cycle studies, IceBac 372 (OD_550_ of ~0.8) was infected with PANV1 or PANV2 (MOI of 10) and incubated aerobically at 5°C. Uninfected culture was used as a control. The numbers of infective free viruses in culture supernatants (Eppendorf table centrifuge, 16,200 × *g*, 5 min, and 4°C) were determined by plaque assay. IceBac 419 and IceBac 430 cells (OD_550_ of ~0.8) were collected (Eppendorf table centrifuge, 16,200 × *g*, 5 min, and 4°C) and resuspended in OANV1 or OANV2 virus stocks, respectively, (MOI of ~8) or in broth (uninfected controls). During the growth, the numbers of free viruses and viable cells in supernatant and pellet fractions (Eppendorf table centrifuge, 16,200 × *g*, 5 min, and 4°C) were determined by plaque assay.

### Virus purification and transmission electron microscopy.

OANV1 was purified from virus stocks by ammonium sulfate precipitation and rate-zonal ultracentrifugation in sucrose using SM buffer as described (24). Particles were negatively stained with uranyl acetate (2% [wt/vol], pH 7) or Nano-W (2% [wt/vol] methylamine tungstate, pH 6.8) prior to transmission electron microscopy (Hitachi HT780 microscope; Electron Microscopy Unit, University of Helsinki). Particle size was measured using ImageJ (67).

### Genome sequencing and annotation.

Nucleic acids were extracted from purified viruses by phenol-ether extraction, precipitated by ethanol-NaCl, sequenced using the Illumina MiSeq platform (DNA Sequencing and Genomics core facility, Helsinki Institute of Life Science, University of Helsinki), and assembled with SPAdes v. 3.9.0 (68).

Geneious Prime 2021.0.2 (https://www.geneious.com) was used for sequence handling. ORFs were predicted using Glimmer, GeneMarkS (Prokaryotic, v. 3.26), MetaGeneAnnotator (http://metagene.nig.ac.jp/), and FGENESV (http://www.softberry.com/berry.phtml?topic=virus&group=programs&subgroup=gfindv). GC content was calculated with Genomics %G~C Content Calculator (https://www.sciencebuddies.org/science-fair-projects/references/genomics-g-c-content-calculator). Transmembrane helices were predicted using TMHMM server v. 2.0 (https://services.healthtech.dtu.dk/service.php?TMHMM-2.0). Whole-genome comparisons for overall nucleotide identity were done with EMBOSS stretcher (69). BLASTN with the whole virus genomes as queries against nonredundant (nr) nucleotide collection (viruses taxid 10239) was used for searching homologous viral genome sequences. Predicted ORFs were assigned with functions based on homology searches with blastx or blastp against the nr protein database (thresholds, E value of 0.00001, query cover 30%, identity 30%) (70), blast conserved domains (E value threshold of 0.01) (71), and HHpred within the Toolkit (E value threshold of 0.01) (72) (searches dated May 2019 to February 2021). VIRFAM was used for putative virus classification based on their neck gene module organization (32). tRNA genes were predicted using tRNAscan-SE v. 2.0 (73).

### Metagenomic analyses.

Virus sequences were searched against Integrated Microbial Genomes/Virus (IMG/VR) database (34) using the whole genomes as queries and blastn search with the maximum E value of 0.00001 (search dated 9 December 2020). Sequence similarities were visualized using Circoletto based on Circos (74), with a blastn search E value threshold of 0.00001. For pairwise sequence comparisons, Easyfig v. 2.2.2 was used (75).

The genome sequences of PANV1, PANV2, OANV1, and OANV2 were compared to the IMG/VR spacer databases (both “isolate” and “metagenome”) (34) using blastn v2.10.0+ (76) with the following parameters: “-dust no -word_size 7.” Only alignments with 0 or 1 mismatch over the entire length of the CRISPR spacer were considered potentially informative hits. The corresponding CRISPR spacer was further examined to filter out low-complexity sequences (e.g., short predicted spacers, including repeat sequences).

### Data availability.

Sequences are available in the GenBank database (MW805361, PANV1; MW805362, PANV2; MW805363, OANV1; MW805364, OANV2).

## References

[1] Vancoppenolle M, Meiners KM, Michel C, Bopp L, Brabant F, Carnat G, Delille B, Lannuzel D, Madec G, Moreau S, Tison J-L, van der Merwe P. 2013. Role of sea ice in global biogeochemical cycles: emerging views and challenges. Quat Sci Rev 79:207–230. doi:10.1016/j.quascirev.2013.04.011.

[2] Dieckmann GS, Hellmer HH. 2010. The importance of sea ice: an overview, p 1–22. *In* Sea Ice. Wiley-Blackwell, Hoboken, NJ.

[3] Arrigo KR. 2014. Sea ice ecosystems. Annu Rev Mar Sci 6:439–467. doi:10.1146/annurev-marine-010213-135103. 24015900

[4] Boetius A, Anesio AM, Deming JW, Mikucki JA, Rapp JZ. 2015. Microbial ecology of the cryosphere: sea ice and glacial habitats. Nat Rev Microbiol 13:677–690. doi:10.1038/nrmicro3522. 26344407

[5] Ewert M, Deming JW. 2013. Sea ice microorganisms: environmental constraints and extracellular responses. Biology (Basel) 2:603–628. doi:10.3390/biology2020603. 24832800 PMC3960889

[6] Maccario L, Sanguino L, Vogel TM, Larose C. 2015. Snow and ice ecosystems: not so extreme. Res Microbiol 166:782–795. doi:10.1016/j.resmic.2015.09.002. 26408452

[7] Thomas D, Dieckmann G. 2002. Antarctic sea ice—a habitat for extremophiles. Science 295:641–644. doi:10.1126/science.1063391. 11809961

[8] Mock T, Thomas DN. 2005. Recent advances in sea‐ice microbiology. Environ Microbiol 7:605–619. doi:10.1111/j.1462-2920.2005.00781.x. 15819843

[9] Chénard C, Lauro FM. 2017. Exploring the viral ecology of high latitude aquatic systems, p 185–200. Microbial Ecology of Extreme Environments, Springer, New York, NY.

[10] López-Bueno A, Tamames J, Velázquez D, Moya A, Quesada A, Alcamí A. 2009. High diversity of the viral community from an Antarctic lake. Science 326:858–861. doi:10.1126/science.1179287. 19892985

[11] Cavicchioli R. 2015. Microbial ecology of Antarctic aquatic systems. Nat Rev Microbiol 13:691–706. doi:10.1038/nrmicro3549. 26456925

[12] Maranger R, Bird DF, Juniper SK. 1994. Viral and bacterial dynamics in Arctic sea ice during the spring algal bloom near Resolute, NWT, Canada. Mar Ecol Prog Ser 111:121–127. doi:10.3354/meps111121.

[13] Gowing MM, Riggs BE, Garrison DL, Gibson AH, Jeffries MO. 2002. Large viruses in Ross Sea late autumn pack ice habitats. Mar Ecol Prog Ser 241:1–11. doi:10.3354/meps241001.

[14] Gowing M. 2003. Large viruses and infected microeukaryotes in Ross Sea summer pack ice habitats. Marine Biology 142:1029–1040. doi:10.1007/s00227-003-1015-x.

[15] Gowing MM, Garrison DL, Gibson AH, Krupp JM, Jeffries MO, Fritsen CH. 2004. Bacterial and viral abundance in Ross Sea summer pack ice communities. Mar Ecol Prog Ser 279:3–12. doi:10.3354/meps279003.

[16] Marchant H, Davidson A, Wright S, Glazebrook J. 2000. The distribution and abundance of viruses in the Southern Ocean during spring. Antarct Sci 12:414–417. doi:10.1017/S0954102000000481.

[17] Wells LE, Deming JW. 2006. Modelled and measured dynamics of viruses in Arctic winter sea‐ice brines. Environ Microbiol 8:1115–1121. doi:10.1111/j.1462-2920.2006.00984.x. 16689732

[18] Säwström C, Lisle J, Anesio AM, Priscu JC, Laybourn-Parry J. 2008. Bacteriophage in polar inland waters. Extremophiles 12:167–175. doi:10.1007/s00792-007-0134-6. 18188502

[19] Brum JR, Hurwitz BL, Schofield O, Ducklow HW, Sullivan MB. 2016. Seasonal time bombs: dominant temperate viruses affect Southern Ocean microbial dynamics. ISME J 10:437–449. doi:10.1038/ismej.2015.125. 26296067 PMC4737935

[20] Anesio AM, Bellas CM. 2011. Are low temperature habitats hot spots of microbial evolution driven by viruses? Trends Microbiol 19:52–57. doi:10.1016/j.tim.2010.11.002. 21130655

[21] Yu Z-C, Chen X-L, Shen Q-T, Zhao D-L, Tang B-L, Su H-N, Wu Z-Y, Qin Q-L, Xie B-B, Zhang X-Y, Yu Y, Zhou B-C, Chen B, Zhang Y-Z. 2015. Filamentous phages prevalent in Pseudoalteromonas spp. confer properties advantageous to host survival in Arctic sea ice. ISME J 9:871–881. doi:10.1038/ismej.2014.185. 25303713 PMC4817708

[22] Borriss M, Helmke E, Hanschke R, Schweder T. 2003. Isolation and characterization of marine psychrophilic phage-host systems from Arctic sea ice. Extremophiles 7:377–384. doi:10.1007/s00792-003-0334-7. 12820036

[23] Luhtanen A-M, Eronen-Rasimus E, Kaartokallio H, Rintala J-M, Autio R, Roine E. 2014. Isolation and characterization of phage–host systems from the Baltic Sea ice. Extremophiles 18:121–130. doi:10.1007/s00792-013-0604-y. 24297705

[24] Luhtanen A-M, Eronen-Rasimus E, Oksanen HM, Tison J-L, Delille B, Dieckmann GS, Rintala J-M, Bamford DH. 2018. The first known virus isolates from Antarctic sea ice have complex infection patterns. FEMS Microbiol Ecol 94:fiy028. doi:10.1093/femsec/fiy028. 29481638

[25] Senčilo A, Luhtanen AM, Saarijärvi M, Bamford DH, Roine E. 2015. Cold‐active bacteriophages from the Baltic Sea ice have diverse genomes and virus–host interactions. Environ Microbiol 17:3628–3641. doi:10.1111/1462-2920.12611. 25156651

[26] Tedesco L, Vichi M. 2014. Sea ice biogeochemistry: a guide for modellers. PLoS One 9:e89217. doi:10.1371/journal.pone.0089217. 24586604 PMC3934902

[27] Adriaenssens EM, Cowan DA. 2014. Using signature genes as tools to assess environmental viral ecology and diversity. Appl Environ Microbiol 80:4470–4480. doi:10.1128/AEM.00878-14. 24837394 PMC4148782

[28] Henthorn KS, Friedman DI. 1995. Identification of related genes in phages phi 80 and P22 whose products are inhibitory for phage growth in Escherichia coli IHF mutants. J Bacteriol 177:3185–3190. doi:10.1128/jb.177.11.3185-3190.1995. 7768817 PMC177009

[29] Casjens SR, Gilcrease EB, Winn-Stapley DA, Schicklmaier P, Schmieger H, Pedulla ML, Ford ME, Houtz JM, Hatfull GF, Hendrix RW. 2005. The generalized transducing Salmonella bacteriophage ES18: complete genome sequence and DNA packaging strategy. J Bacteriol 187:1091–1104. doi:10.1128/JB.187.3.1091-1104.2005. 15659686 PMC545730

[30] Szewczyk B, Bienkowska-Szewczyk K, Kozloff LM. 1986. Identification of T4 gene 25 product, a component of the tail baseplate, as a 15K lysozyme. Mol Gen Genet 202:363–367. doi:10.1007/BF00333263. 3520236

[31] Petrov VM, Ratnayaka S, Nolan JM, Miller ES, Karam JD. 2010. Genomes of the T4-related bacteriophages as windows on microbial genome evolution. Virol J 7:292. doi:10.1186/1743-422X-7-292. 21029436 PMC2993671

[32] Lopes A, Tavares P, Petit M-A, Guérois R, Zinn-Justin S. 2014. Automated classification of tailed bacteriophages according to their neck organization. BMC Genomics 15:1027. doi:10.1186/1471-2164-15-1027. 25428721 PMC4362835

[33] Kavagutti VS, Andrei A-Ş, Mehrshad M, Salcher MM, Ghai R. 2019. Phage-centric ecological interactions in aquatic ecosystems revealed through ultra-deep metagenomics. Microbiome 7:135. doi:10.1186/s40168-019-0752-0. 31630686 PMC6802176

[34] Roux S, Páez-Espino D, Chen I-MA, Palaniappan K, Ratner A, Chu K, Reddy TBK, Nayfach S, Schulz F, Call L, Neches RY, Woyke T, Ivanova NN, Eloe-Fadrosh EA, Kyrpides NC. 2021. IMG/VR v3: an integrated ecological and evolutionary framework for interrogating genomes of uncultivated viruses. Nucleic Acids Res 49:D764–D775. doi:10.1093/nar/gkaa946. 33137183 PMC7778971

[35] Panwar P, Allen MA, Williams TJ, Hancock AM, Brazendale S, Bevington J, Roux S, Páez-Espino D, Nayfach S, Berg M, Schulz F, Chen I-MA, Huntemann M, Shapiro N, Kyrpides NC, Woyke T, Eloe-Fadrosh EA, Cavicchioli R. 2020. Influence of the polar light cycle on seasonal dynamics of an Antarctic lake microbial community. Microbiome 8:116. doi:10.1186/s40168-020-00889-8. 32772914 PMC7416419

[36] Hawley ER, Malfatti SA, Pagani I, Huntemann M, Chen A, Foster B, Copeland A, del Rio TG, Pati A, Jansson JR, Gilbert JA, Tringe SG, Lorenson TD, Hess M. 2014. Metagenomes from two microbial consortia associated with Santa Barbara seep oil. Mar Genomics 18:97–99. doi:10.1016/j.margen.2014.06.003. 24958360

[37] Hawley ER, Piao H, Scott NM, Malfatti S, Pagani I, Huntemann M, Chen A, Glavina Del Rio T, Foster B, Copeland A, Jansson J, Pati A, Tringe S, Gilbert JA, Lorenson TD, Hess M. 2014. Metagenomic analysis of microbial consortium from natural crude oil that seeps into the marine ecosystem offshore Southern California. Stand Genomic Sci 9:1259–1274. doi:10.4056/sigs.5029016. 25197496 PMC4149020

[38] Sun M, Zhan Y, Marsan D, Páez-Espino D, Cai L, Chen F. 2021. Uncultivated viral populations dominate estuarine viromes on the spatiotemporal scale. mSystems 6:e01020-20. doi:10.1128/mSystems.01020-20. 33727395 PMC8546989

[39] Tschitschko B, Erdmann S, DeMaere MZ, Roux S, Panwar P, Allen MA, Williams TJ, Brazendale S, Hancock AM, Eloe-Fadrosh EA, Cavicchioli R. 2018. Genomic variation and biogeography of Antarctic haloarchaea. Microbiome 6:113. doi:10.1186/s40168-018-0495-3. 29925429 PMC6011602

[40] Williams TJ, Allen MA, Ivanova N, Huntemann M, Haque S, Hancock AM, Brazendale S, Cavicchioli R. 2021. Genome analysis of a verrucomicrobial endosymbiont with a tiny genome discovered in an Antarctic lake. Front Microbiol 12:674758. doi:10.3389/fmicb.2021.674758. 34140946 PMC8204192

[B41] Ji X, Yu H, Zhang Q, Lin L, Wei Y. 2015. Isolation and characterization of a novel lytic cold-active bacteriophage VNPH-1 from the Napahai wetland in China. Ann Microbiol 65:1789–1796. doi:10.1007/s13213-014-1018-5.

[B42] Ji X, Zhang C, Fang Y, Zhang Q, Lin L, Tang B, Wei Y. 2015. Isolation and characterization of glacier VMY22, a novel lytic cold-active bacteriophage of Bacillus cereus. Virol Sin 30:52–58. doi:10.1007/s12250-014-3529-4. 25680445 PMC8200904

[43] Li M, Wang J, Zhang Q, Lin L, Kuang A, Materon LA, Ji X, Wei Y. 2016. Isolation and characterization of the lytic cold-active bacteriophage MYSP06 from the Mingyong Glacier in China. Curr Microbiol 72:120–127. doi:10.1007/s00284-015-0926-3. 26500034

[44] Qin K, Ji X, Zhang C, Ding Y, Kuang A, Zhang S, Zhang Q, Lin L, Wei Y. 2017. Isolation and characterization of wetland VSW-3, a novel lytic cold-active bacteriophage of Pseudomonas fluorescens. Can J Microbiol 63:110–118. doi:10.1139/cjm-2016-0368. 28001438

[B45] Xiang Y, Wang S, Li J, Wei Y, Zhang Q, Lin L, Ji X. 2018. Isolation and characterization of two lytic cold-active bacteriophages infecting Pseudomonas fluorescens from the Napahai plateau wetland. Can J Microbiol 64:183–190. doi:10.1139/cjm-2017-0572. 29253355

[46] Wells LE, Deming JW. 2006. Effects of temperature, salinity and clay particles on inactivation and decay of cold-active marine Bacteriophage 9A. Aquat Microb Ecol 45:31–39. doi:10.3354/ame045031.

[47] Kukkaro P, Bamford DH. 2009. Virus–host interactions in environments with a wide range of ionic strengths. Environ Microbiol Rep 1:71–77. doi:10.1111/j.1758-2229.2008.00007.x. 23765723

[48] Rodela ML, Sabet S, Peterson A, Dillon JG. 2019. Broad environmental tolerance for a Salicola host-phage pair isolated from the Cargill Solar Saltworks, Newark, CA, USA. Microorganisms 7:106. doi:10.3390/microorganisms7040106. PMC6518143 31010175

[49] Aalto AP, Bitto D, Ravantti JJ, Bamford DH, Huiskonen JT, Oksanen HM. 2012. Snapshot of virus evolution in hypersaline environments from the characterization of a membrane-containing Salisaeta icosahedral phage 1. Proc Natl Acad Sci USA 109:7079–7084. doi:10.1073/pnas.1120174109. 22509017 PMC3344969

[50] Jończyk E, Kłak M, Międzybrodzki R, Górski A. 2011. The influence of external factors on bacteriophages—review. Folia Microbiol (Praha) 56:191–200. doi:10.1007/s12223-011-0039-8. 21625877 PMC3131515

[51] Vörös Z, Csík G, Herényi L, Kellermayer M. 2018. Temperature-dependent nanomechanics and topography of bacteriophage T7. J Virol 92:e01236-18. doi:10.1128/JVI.01236-18. 30089696 PMC6158431

[52] Langlet J, Gaboriaud F, Gantzer C. 2007. Effects of pH on plaque forming unit counts and aggregation of MS2 bacteriophage. J Appl Microbiol 103:1632–1638. doi:10.1111/j.1365-2672.2007.03396.x. 17953574

[53] Szermer-Olearnik B, Drab M, Mąkosa M, Zembala M, Barbasz J, Dąbrowska K, Boratyński J. 2017. Aggregation/dispersion transitions of T4 phage triggered by environmental ion availability. J Nanobiotechnol 15:32. doi:10.1186/s12951-017-0266-5. PMC5404661 28438164

[54] Liu Y, Wang J, Liu Y, Wang Y, Zhang Z, Oksanen HM, Bamford DH, Chen X. 2015. Identification and characterization of SNJ 2, the first temperate pleolipovirus integrating into the genome of the SNJ 1‐lysogenic archaeal strain. Mol Microbiol 98:1002–1020. doi:10.1111/mmi.13204. 26331239

[55] Laybourn-Parry J, Marshall WA, Madan NJ. 2007. Viral dynamics and patterns of lysogeny in saline Antarctic lakes. Polar Biol 30:351–358. doi:10.1007/s00300-006-0191-9.

[56] Säwström C, Anesio MA, Granéli W, Laybourn-Parry J. 2007. Seasonal viral loop dynamics in two large ultraoligotrophic Antarctic freshwater lakes. Microb Ecol 53:1–11. doi:10.1007/s00248-006-9146-5. 17075732

[57] Mäntynen S, Laanto E, Oksanen HM, Poranen MM, Díaz-Muñoz SL. 2021. Black box of phage–bacterium interactions: exploring alternative phage infection strategies. Open Biol 11:210188. doi:10.1098/rsob.210188. 34520699 PMC8440029

[58] Middelboe M, Nielsen TG, Bjørnsen PK. 2002. Viral and bacterial production in the North Water: in situ measurements, batch-culture experiments and characterization and distribution of a virus–host system. Deep Sea Res II: Top Stud Oceanogr 49:5063–5079. doi:10.1016/S0967-0645(02)00178-9.

[59] Delisle A, Levin R. 1972. Effect of temperature on an obligately psychrophilic phage-host system of Pseudomonas putrefaciens. Antonie Van Leeuwenhoek 38:9–15. doi:10.1007/BF02328072. 4537090

[60] Wells LE, Deming JW. 2006. Characterization of a cold-active bacteriophage on two psychrophilic marine hosts. Aquat Microb Ecol 45:15–29. doi:10.3354/ame045015.

[61] Sillankorva S, Oliveira R, Vieira MJ, Sutherland I, Azeredo J. 2004. Pseudomonas fluorescens infection by bacteriophage ΦS1: the influence of temperature, host growth phase and media. FEMS Microbiol Lett 241:13–20. doi:10.1016/j.femsle.2004.06.058. 15556704

[62] Brum JR, Ignacio-Espinoza JC, Roux S, Doulcier G, Acinas SG, Alberti A, Chaffron S, Cruaud C, de Vargas C, Gasol JM, Gorsky G, Gregory AC, Guidi L, Hingamp P, Iudicone D, Not F, Ogata H, Pesant S, Poulos BT, Schwenck SM, Speich S, Dimier C, Kandels-Lewis S, Picheral M, Searson S, Bork P, Bowler C, Sunagawa S, Wincker P, Karsenti E, Sullivan MB, Tara Oceans Coordinators. 2015. Patterns and ecological drivers of ocean viral communities. Science 348:1261498. doi:10.1126/science.1261498. 25999515

[63] Colangelo-Lillis JR, Deming JW. 2013. Genomic analysis of cold-active Colwelliaphage 9A and psychrophilic phage–host interactions. Extremophiles 17:99–114. doi:10.1007/s00792-012-0497-1. 23224375

[64] Brum JR, Sullivan MB. 2015. Rising to the challenge: accelerated pace of discovery transforms marine virology. Nat Rev Microbiol 13:147–159. doi:10.1038/nrmicro3404. 25639680

[65] Hurwitz BL, Sullivan MB. 2013. The Pacific Ocean Virome (POV): a marine viral metagenomic dataset and associated protein clusters for quantitative viral ecology. PLoS One 8:e57355. doi:10.1371/journal.pone.0057355. 23468974 PMC3585363

[66] Adams MH. 1959. Bacteriophages. Interscience Publishers, New York, NY.

[67] Schneider CA, Rasband WS, Eliceiri KW. 2012. NIH Image to ImageJ: 25 years of image analysis. Nat Methods 9:671–675. doi:10.1038/nmeth.2089. 22930834 PMC5554542

[68] Bankevich A, Nurk S, Antipov D, Gurevich AA, Dvorkin M, Kulikov AS, Lesin VM, Nikolenko SI, Pham S, Prjibelski AD, Pyshkin AV, Sirotkin AV, Vyahhi N, Tesler G, Alekseyev MA, Pevzner PA. 2012. SPAdes: a new genome assembly algorithm and its applications to single-cell sequencing. J Comput Biol 19:455–477. doi:10.1089/cmb.2012.0021. 22506599 PMC3342519

[69] Madeira F, Park YM, Lee J, Buso N, Gur T, Madhusoodanan N, Basutkar P, Tivey ARN, Potter SC, Finn RD, Lopez R. 2019. The EMBL-EBI search and sequence analysis tools APIs in 2019. Nucleic Acids Res 47:W636–W641. doi:10.1093/nar/gkz268. 30976793 PMC6602479

[70] Altschul SF, Gish W, Miller W, Myers EW, Lipman DJ. 1990. Basic local alignment search tool. J Mol Biol 215:403–410. doi:10.1016/S0022-2836(05)80360-2. 2231712

[71] Lu S, Wang J, Chitsaz F, Derbyshire MK, Geer RC, Gonzales NR, Gwadz M, Hurwitz DI, Marchler GH, Song JS, Thanki N, Yamashita RA, Yang M, Zhang D, Zheng C, Lanczycki CJ, Marchler-Bauer A. 2020. CDD/SPARCLE: the conserved domain database in 2020. Nucleic Acids Res 48:D265–D268. doi:10.1093/nar/gkz991. 31777944 PMC6943070

[72] Zimmermann L, Stephens A, Nam S-Z, Rau D, Kübler J, Lozajic M, Gabler F, Söding J, Lupas AN, Alva V. 2018. A completely reimplemented MPI bioinformatics toolkit with a new HHpred server at its core. J Mol Biol 430:2237–2243. doi:10.1016/j.jmb.2017.12.007. 29258817

[73] Chan PP, Lowe TM. 2019. tRNAscan-SE: searching for tRNA genes in genomic sequences. Methods Mol Biol 1962:1–14. doi:10.1007/978-1-4939-9173-0_1. 31020551 PMC6768409

[74] Darzentas N. 2010. Circoletto: visualizing sequence similarity with Circos. Bioinformatics 26:2620–2621. doi:10.1093/bioinformatics/btq484. 20736339

[75] Sullivan MJ, Petty NK, Beatson SA. 2011. Easyfig: a genome comparison visualizer. Bioinformatics 27:1009–1010. doi:10.1093/bioinformatics/btr039. 21278367 PMC3065679

[76] Camacho C, Coulouris G, Avagyan V, Ma N, Papadopoulos J, Bealer K, Madden TL. 2009. BLAST+: architecture and applications. BMC Bioinformatics 10:421. doi:10.1186/1471-2105-10-421. 20003500 PMC2803857

