# Supplementary material for: Virus-Host Interactions and Genetic Diversity of Antarctic Sea Ice Bacteriophages
Source: mBio. 2022 May 9;13(3):e00651-22. doi: 10.1128/mbio.00651-22 (PMC9239159; doi:10.1128/mbio.00651-22)
Supplement: FIG S1 [file mbio.00651-22-s0006.pdf]

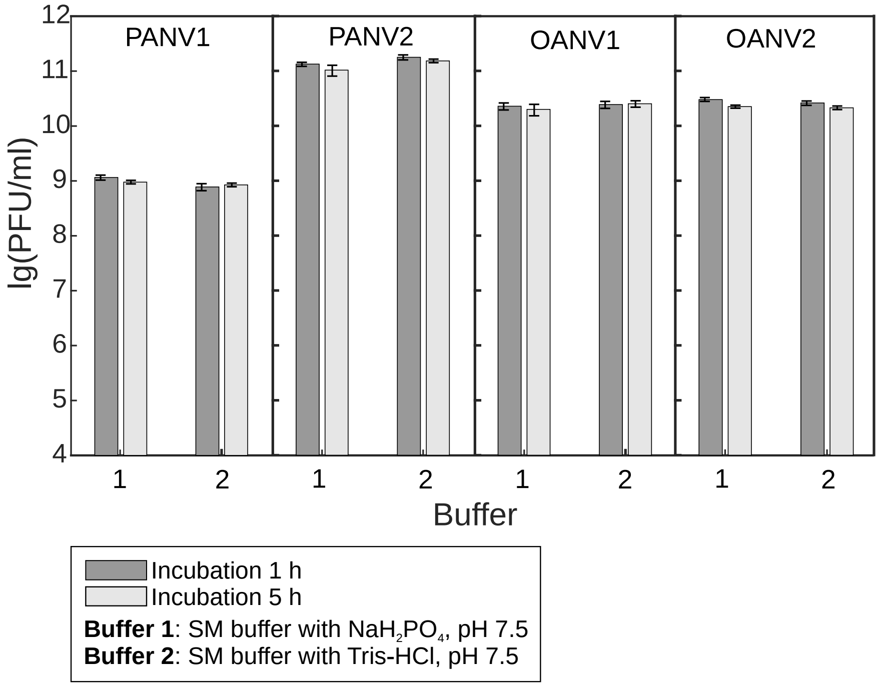

Figure S1. Stability of virus infectivity in SM buffer containing NaH_2PO_4 (buffer 1) or Tris-HCl (buffer 2), pH 7.5. Virus stocks were diluted 1000-fold in the buffers and incubated 1 h (dark grey) and 5 h (light grey). Bars represent means of at least three independent replicates with standard error of the mean shown as error bars.
