# Supplementary material for: Virus-Host Interactions and Genetic Diversity of Antarctic Sea Ice Bacteriophages
Source: mBio. 2022 May 9;13(3):e00651-22. doi: 10.1128/mbio.00651-22 (PMC9239159; doi:10.1128/mbio.00651-22)
Supplement: TABLE S1 [file mbio.00651-22-s0001.pdf]

Table S1. Putative functions assigned to PANV1 ORF products.

ORF	Start, nt	Stop, nt	Direction[a]	Gene product (gp)	Protein size, aa	TMHs[b]	Putative function	Best Blastx match (thresholds: E-value 1e-5, query cover 30%, identity 30%, search dated 17.02.2021)	
								**Protein [organism], accession number**	**Query cover/identity, %, E-value**
ORF1	1	450	F	gp1	149	0	Terminase small subunit	small terminase [Klebsiella pneumoniae], WP_153932317.1	96/43, 2e-31
ORF2	440	2 257	F	gp2	605	1	Terminase large subunit	putative terminase DNA packaging enzyme large subunit [Cronobacter phage S13], YP_009196472.1	92/53, 0
ORF3	2 350	3 858	F	gp3	502	0	Tail sheath protein	tail sheath protein [Klebsiella phage KPN5], QEG11335.1	99/47, 5e-115
ORF4	3 872	4 372	F	gp4	166	0	Major tail protein (tail tube protein)	tail tube protein [Escherichia phage JS98], YP_001595295.1	96/59, 1e-63
ORF5	4 379	5 917	F	gp5	512	0	Head portal vertex protein	putative head portal vertex protein [Cronobacter phage S13], YP_009196469.1	98/46, 1e-134
ORF6	5 907	6 086	F	gp6	59	0		-[c]	-
ORF7	6 079	6 372	F	gp7	97	0	Prohead core protein	-	-
ORF8	6 388	6 972	F	gp8	194	0		hypothetical protein BV459_06015 [Thermoplasmata archaeon M11B2D], PNX46619.1	82/43, 3e-30
ORF9	6 974	7 798	F	gp9	274	0	Prohead core scaffold protein and protease	prohead core protein protease [Vibrio phage VH7D], YP_009006289.1	65/46, 9e-41
ORF10	7 805	8 146	F	gp10	113	0		-	-
ORF11	8 279	9 106	F	gp11	275	0	Prohead core scaffold protein	prohead core protein [Acidobacteria bacterium], MBF84619.1	78/43, 8e-37
ORF12	9 130	10 764	F	gp12	544	0	Major capsid protein	major head protein [Vibrio phage nt-1], YP_008125183.1	98/51, 2e-174
ORF13	10 825	11 475	R	gp13	216	0	Endonuclease	hypothetical protein [Campylobacter coli], EDO6940290.1	56/43, 1e-12
ORF14	11 685	12 830	R	gp14	381	0		-	-
ORF15	12 893	14 371	F	gp15	492	0	DNA helicase	DNA helicase [Providencia phage PSTCR6], QPB12403.1	97/45, 9e-132
ORF16	14 368	14 556	F	gp16	62	0		-	-

ORF17	14 553	14 768	R	gp17	71	0		hypothetical protein [Gammaproteobacteria bacterium], NBT29188.1	94/57, 3e-18
ORF18	14 824	15 303	R	gp18	159	0		hypothetical protein B0D91_10585 [Oceanospirillales bacterium LUC14_002_19_P2], OQX35588.1	96/41, 2e-30
ORF19	15 316	15 483	R	gp19	55	1		-	-
ORF20	15 483	16 133	R	gp20	216	0		hypothetical protein [Aeromonas veronii], WP_201997786.1	39/38, 3e-8
ORF21	16 126	16 317	R	gp21	63	0		-	-
ORF22	16 317	16 574	R	gp22	85	0		hypothetical protein AXW14_08595 [Alteromonas sp. Nap_26], KXJ61659.1	76/46, 6e-12
ORF23	16 610	16 858	R	gp23	82	0		-	-
ORF24	16 858	17 175	R	gp24	105	0		-	-
ORF25	17 175	17 483	R	gp25	102	0		-	-
ORF26	17 487	18 035	R	gp26	182	0	Dihydrofolate reductase	dihydrofolate reductase [Vibrio phage vB_VpS_PG07], YP_009808539.1	81/42, 3e-28
ORF27	18 037	18 357	R	gp27	106	0		hypothetical protein A3F91_09710 [Flavobacteria bacterium RIFCSPLOWO2_12_FULL_35_11], OGS70779.1	89/42, 4e-16
ORF28	18 354	18 713	R	gp28	119	0	DNA processing protein	DUF2493 domain-containing protein [Bacteroidales bacterium], MBE6229754.1	96/52, 4e-30
ORF29	18 710	19 120	R	gp29	136	0	Recombination protein	hypothetical protein [Candidatus Pelagibacter sp.], MAJ66456.1	71/31, 2e-9
ORF30	19 459	20 154	F	gp30	231	0	Deoxynucleoside monophosphate kinase	gp1 dNMP kinase [Acinetobacter phage Acj61], YP_004009770.1	99/36, 4e-34
ORF31	20 162	20 545	F	gp31	127	0		-	-
ORF32	20 597	20 794	F	gp32	65	0		-	-
ORF33	20 801	21 166	F	gp33	121	0		-	-
ORF34	21 163	21 630	F	gp34	155	0		-	-

ORF35	21 660	22 283	F	gp35	207	0	Permuted papain-like amidase	hypothetical protein [Ectothiorhodospiraceae bacterium WFHF3C12], MBA1147110.1	92/33, 8e-21
ORF36	22 684	23 010	F	gp36	108	0	Phospholipase	DUF1353 domain-containing protein [Shewanella sp. WE21], WP_105251766.1	98/36, 4e-18
ORF37	23 076	23 522	F	gp37	148	0	NADAR (NAD and ADP-ribose) superfamily protein	hypothetical protein KLPPOU148_023 [Klebsiella phage vB_KpnM_15-38_KLPPOU148], QGZ13401.1	94/49, 6e-38
ORF38	23 559	23 726	F	gp38	55	0		-	-
ORF39	23 994	24 137	F	gp39	47	0		-	-
ORF40	24 223	24 342	F	gp40	39	0		-	-
ORF41	24 339	24 767	F	gp41	142	0		-	-
ORF42	24 784	25 194	F	gp42	136	0		hypothetical protein [uncultured Mediterranean phage], ANS04486.1	99/50, 3e-38
ORF43	25 315	25 599	F	gp43	94	0		-	-
ORF44	25 612	25 845	F	gp44	77	0		-	-
ORF45	25 842	26 114	F	gp45	90	0		-	-
ORF46	26 111	26 476	F	gp46	121	0		-	-
ORF47	26 508	26 621	F	gp47	37	0		-	-
ORF48	26 618	27 196	F	gp48	192	0		DUF3293 domain-containing protein [Proteobacteria bacterium], MBC8442968.1	99/45, 5e-38
ORF49	27 224	27 550	F	gp49	108	0		-	-
ORF50	27 547	28 014	F	gp50	155	0		-	-
ORF51	27 995	28 303	R	gp51	102	0	Mechanosensitive channel (membrane protein)	hypothetical protein CBC05_09310 [Flavobacteriales bacterium TMED45], OUU16045.1	88/40, 1e-12
ORF52	28 351	28 581	F	gp52	76	0		-	-
ORF53	28 633	28 884	F	gp53	83	0		-	-
ORF54	28 888	30 045	F	gp54	385	0	DNA ligase	ATP-dependent DNA ligase [Vibrio phage RYC], BAV80838.1	94/42, 3e-99
ORF55	30 056	31 117	F	gp55	353	0	RNA ligase	hypothetical protein [Haliea sp.], MBM68177.1	98/40, 9e-64

ORF56	31 241	31 735	F	gp56	164	0	RNA polymerase sigma factor	sigma factor for late transcription [Citrobacter phage Merlin], YP_009203779.1	90/45, 5e-23
ORF57	31 732	31 956	F	gp57	74	0		-	-
ORF58	31 943	32 113	F	gp58	56	0		-	-
ORF59	32 132	33 157	F	gp59	341	0	Nuclease	putative recombination endonuclease subunit [Escherichia phage HY01], YP_009148506.1	97/39, 1e-66
ORF60	33 157	35 346	F	gp60	729	0	Nuclease	recombination endonuclease subunit [Vibrio phage KVP40], NP_899322.1	99/36, 1e-128
ORF61	35 449	35 736	F	gp61	95	0		-	-
ORF62	35 740	36 399	F	gp62	219	0	Sliding clamp DNA polymerase accessory protein	sliding clamp DNA polymerase accessory protein [Prochlorococcus phage P-SSM2], YP_214389.1	99/41, 6e-47
ORF63	36 678	37 625	F	gp63	315	0	DNA polymerase clamp loader subunit	DNA polymerase accessory protein 44 [Vibrio phage VH7D], YP_009006372.1	98/46, 1e-94
ORF64	37 622	38 077	F	gp64	151	0	DNA polymerase clamp loader subunit	polymerase accessory protein 62 [Vibrio phage VH7D], YP_009006373.1	80/49, 3e-28
ORF65	38 128	38 541	F	gp65	137	0	Translational repressor	RegA translational repressor protein [Vibrio phage nt-1], YP_008125278.1	87/63, 7e-49
ORF66	38 548	38 730	F	gp66	60	0	Transcriptional regulator	-	-
ORF67	38 814	39 260	F	gp67	148	0		hypothetical protein KQ78_02144 [Candidatus Izimaplasma sp. HR2], KFZ25651.1	86/49, 4e-26
ORF68	39 277	40 044	F	gp68	255	0	DNA adenine methylase	hypothetical protein [Haliea sp.], MBM68201.1	98/36, 4e-38
ORF69	40 046	41 533	F	gp69	495	0	DNA polymerase	DNA-directed DNA polymerase [Vibrio phage V09], QIW90952.1	97/46, 1e-131
ORF70	41 530	41 871	F	gp70	113	1		-	-
ORF71	41 846	42 316	F	gp71	156	0		-	-
ORF72	42 373	42 639	F	gp72	88	2		-	-
ORF73	42 623	43 042	F	gp73	139	1		-	-
ORF74	43 029	43 289	F	gp74	86	1		-	-

ORF75	43 298	44 383	F	gp75	361	0	DNA polymerase	putative DNA polymerase [Cronobacter phage S13], YP_009196632.1	97/50, 2e-99
ORF76	44 445	45 515	F	gp76	356	0	Recombinase	UvsX [Escherichia phage vB_EcoM_011D4], QMP82623.1	91/60, 2e-128
ORF77	45 508	45 774	F	gp77	88	0		-	-
ORF78	45 800	47 170	F	gp78	456	0	Replicative DNA helicase	41 helicase [Vibrio phage KVP40], NP_899258.1	94/57, 4e-165
ORF79	47 183	48 469	F	gp79	428	0	DNA helicase	DNA helicase [Vibrio phage henriette 12B8], YP_007878016.1	94/41, 6e-95
ORF80	48 466	49 707	F	gp80	413	0	Glycosyltransferase	hypothetical protein ST65p128 [Aeromonas virus 65], YP_004300965.1	92/55, 9e-131
ORF81	49 776	50 366	F	gp81	196	0		-	-
ORF82	50 416	50 751	F	gp82	111	0		-	-
ORF83	50 744	51 031	F	gp83	95	0		-	-
ORF84	51 031	51 225	F	gp84	64	0		-	-
ORF85	51 227	51 634	F	gp85	135	0		hypothetical protein [Myoviridae sp.], QHJ78821.1	65/39, 1e-6
ORF86	51 718	52 509	F	gp86	263	0		hypothetical protein [Acidobacteria bacterium], MBF84499.1	96/42, 1e-55
ORF87	52 513	52 812	F	gp87	99	0		-	-
ORF88	52 812	53 003	F	gp88	63	0		-	-
ORF89	53 022	53 486	F	gp89	154	0	RNA phosphodiesterase, RNA ligase	hypothetical protein COA52_01050 [Rhizobiales bacterium], PCJ96827.1	80/31, 3e-11
ORF90	53 486	53 926	F	gp90	146	0	Nuclease	hypothetical protein [Ralstonia phage RSP15], YP_009277031.1	95/36, 3e-27
ORF91	53 966	54 370	F	gp91	134	0		-	-
ORF92	54 516	55 265	F	gp92	249	0	RelA/SpoT family protein, GTP pyrophosphokinase	RelA/SpoT family protein [Sinorhizobium phage phiM12], YP_009143198.1	57/38, 2e-16
ORF93	55 330	55 605	F	gp93	91	0		-	-
ORF94	55 645	56 055	F	gp94	136	0	RelA/SpoT family protein, GTP pyrophosphokinase	HD domain-containing protein [Streptococcus mitis], WP_125386451.1	97/49, 5e-32
ORF95	56 071	57 072	F	gp95	333	0	DNA primase	DNA primase [Aeromonas phage CC2], YP_007010267.1	99/39, 1e-68

ORF96	57 076	57 300	F	gp96	74	0		hypothetical protein [Rheinheimera pacifica], WP_092791076.1	70/55, 5e-13
ORF97	57 300	57 821	F	gp97	173	0	dUTPase	dCTP pyrophosphatase [Shewanella phage Thanatos-1], QJT71759.1	99/54, 7e-52
ORF98	57 866	58 531	F	gp98	221	0	Exonuclease	exonuclease A [Shigella phage SP18], YP_003934639.1	96/40, 9e-39
ORF99	58 537	58 752	F	gp99	71	0		-	-
ORF100	58 763	59 254	F	gp100	163	0	Dephospho-CoA kinase	hypothetical protein [Pseudomonas nitritireducens], WP_184596448.1	85/60, 7e-58
ORF101	59 258	59 449	F	gp101	63	2		-	-
ORF102	59 468	59 932	F	gp102	154	0		-	-
ORF103	59 929	61 722	F	gp103	597	0	DNA topoisomerase large subunit	topoisomerase II large subunit [Vibrio phage nt-1], YP_008125243.2	99/53, 0
ORF104	61 771	63 555	F	gp104	594	0		hypothetical protein [Polaribacter sp. SA4-12], WP_087519593.1	81/29, 3e-26
ORF105	63 565	65 103	F	gp105	512	0		hypothetical protein DBP02_15130 [gamma proteobacterium symbiont of Ctena orbiculata], PUB82384.1	99/67, 0
ORF106	65 103	65 408	F	gp106	101	0		hypothetical protein DRQ35_05515 [Gammaproteobacteria bacterium], RKZ78721.1	99/38, 2e-14
ORF107	65 405	66 055	F	gp107	216	0		hypothetical protein DRR06_12840 [Gammaproteobacteria bacterium], RLA43131.1	60/73, 2e-61
ORF108	66 052	66 498	F	gp108	148	0		-	-
ORF109	66 461	66 889	F	gp109	142	0		-	-
ORF110	66 925	67 167	F	gp110	80	2		-	-
ORF111	67 171	67 500	F	gp111	109	0		-	-
ORF112	67 497	67 832	F	gp112	111	0	Ribonucleotide reductase	hypothetical protein [Candidatus Brocadiales bacterium], MBC8552156.1	92/42, 4e-12
ORF113	67 829	69 481	F	gp113	550	0	DNA transfer (T5 phage A1-like) protein	hypothetical protein FDG98_gp109 [Pseudomonas phage pf16], YP_009595642.1	98/49, 0
ORF114	69 698	70 243	F	gp114	181	0		hypothetical protein PBI_SCTP2_304 [Salicola phage SCTP-2], ASV44319.1	69/41, 6e-22

ORF115	70 479	71 762	F	gp115	427	0	DNA gyrase/topoisomerase	DNA topoisomerase medium subunit [Vibrio phage VH7D], YP_009006242.1	98/46, 3e-131
ORF116	71 842	72 390	F	gp116	182	0	SprT-like domain-containing protein, metalloprotease	SprT Zn-dependent metalloprotease, SprT family [uncultured Caudovirales phage], CAB4122749.1	91/33, 2e-13
ORF117	72 381	72 599	F	gp117	72	0		-	-
ORF118	72 589	72 852	F	gp118	87	0		hypothetical protein COB22_05905 [Cycloclasticus sp.], PHS71950.1	79/55, 8e-17
ORF119	72 849	73 166	F	gp119	105	0		-	-
ORF120	73 166	73 426	F	gp120	86	0		hypothetical protein AMS21_00950 [Gemmatimonas sp. SG8_38_2], KPK66753.1	96/48, 9e-18
ORF121	73 505	73 669	F	gp121	54	0		-	-
ORF122	73 756	74 334	F	gp122	192	0		hypothetical protein [Rhodospirillaceae bacterium], MAL73386.1	99/34, 1e-21
ORF123	74 441	74 875	F	gp123	144	0		-	-
ORF124	74 875	74 988	F	gp124	37	1		-	-
ORF125	74 992	75 363	F	gp125	123	2		-	-
ORF126	75 449	75 868	F	gp126	139	0		-	-
ORF127	75 868	76 131	F	gp127	87	0		hypothetical protein [Flavobacteriaceae bacterium], MBK43679.1	86/39, 5e-11
ORF128	76 131	76 376	F	gp128	81	0	Co-translational protein	-	-
ORF129	76 373	76 549	F	gp129	58	2		-	-
ORF130	76 546	77 268	F	gp130	240	0	Methylase	hypothetical protein [Thiomicrospira sp.], NCN66377.1	97/53, 1e-75
ORF131	77 346	77 720	F	gp131	124	0		-	-
ORF132	77 704	78 156	F	gp132	150	0		-	-
ORF133	78 157	78 399	F	gp133	80	0	Anti-sigma factor	anti-sigma70 protein [Aeromonas phage AS-zj], YP_009834612.1	91/47, 2e-16
ORF134	78 396	78 776	F	gp134	126	0		-	-
ORF135	78 785	79 015	F	gp135	76	0		-	-
ORF136	79 012	80 643	R	gp136	543	0		-	-
ORF137	80 708	84 376	R	gp137	1222	0		-	-
ORF138	84 425	85 558	R	gp138	377	0		-	-

ORF139	85 613	89 332	R	gp139	1239	0		-	-
ORF140	89 354	90 508	R	gp140	384	0		-	-
ORF141	90 518	90 883	R	gp141	121	0		-	-
ORF142	90 977	91 900	F	gp142	307	0	Ribonuclease H	ribonuclease H [Vibrio phage VH7D], YP_009006295.1	99/38, 1e-63
ORF143	91 971	92 231	F	gp143	86	0	double-stranded DNA-binding protein	Double-stranded DNA-binding protein [uncultured Caudovirales phage], CAB4124338.1	94/39, 3e-15
ORF144	92 234	92 479	F	gp144	81	0	RNA-polymerase-associated protein, transcription coactivator	-	-
ORF145	92 506	93 153	F	gp145	215	0	DNA helicase loader	helicase loading protein [Acidobacteria bacterium], MBF84553.1	82/30, 7e-16
ORF146	93 176	94 063	F	gp146	295	0	Single-stranded DNA binding protein	single stranded DNA-binding protein [Vibrio phage phi-Grn1], ALP47019.1	79/54, 5e-83
ORF147	94 143	96 587	F	gp147	814	0	Ribonucleoside reductase alpha subunit	hypothetical protein HWC27_gp186 [Aeromonas phage 4_L372D], YP_009846753.1	88/44, 0
ORF148	96 637	97 731	F	gp148	364	1	Ribonucleoside reductase beta subunit	ribonucleoside diphosphate reductase subunit beta [Vibrio phage eugene 12A10], YP_009223038.1	99/37, 8e-75
ORF149	97 810	97 947	F	gp149	45	0		-	-
ORF150	97 944	98 129	F	gp150	61	0		hypothetical protein COA43_14680 [Robiginitomaculum sp.], PHR55961.1	98/36, 5e-6
ORF151	98 139	98 420	F	gp151	93	0		-	-
ORF152	98 407	98 832	F	gp152	141	0		-	-
ORF153	98 832	99 155	F	gp153	107	0		-	-
ORF154	99 152	99 502	F	gp154	116	0		hypothetical protein GOVbin8609_64 [Prokaryotic dsDNA virus sp.], QDP55932.1	99/48, 2e-35
ORF155	99 499	99 723	F	gp155	74	0		-	-
ORF156	99 720	99 869	F	gp156	49	0		-	-
ORF157	99 935	100 147	F	gp157	70	0		-	-
ORF158	100 149	100 394	F	gp158	81	0		-	-
ORF159	100 391	100 681	F	gp159	96	0		-	-

ORF160	100 678	101 079	F	gp160	133	0		hypothetical protein Rostov7_00011 [Vibrio phage Rostov 7], QBJ00996.1	88/35, 2e-9
ORF161	101 076	101 306	F	gp161	76	0		-	-
ORF162	101 303	101 656	F	gp162	117	0		hypothetical protein COB09_18935 [Thalassobium sp.], PHS61024.1	78/63, 3e-35
ORF163	101 634	101 936	F	gp163	100	0		-	-
ORF164	101 917	102 144	F	gp164	75	0		-	-
ORF165	102 144	102 326	F	gp165	60	0		-	-
ORF166	102 414	102 614	F	gp166	66	0		hypothetical protein [Idiomarinaceae bacterium], MBG23687.1	97/58, 1e-12
ORF167	102 611	102 853	F	gp167	80	0		-	-
ORF168	102 913	103 125	F	gp168	70	0		-	-
ORF169	103 118	103 285	F	gp169	55	0		-	-
ORF170	103 366	103 689	F	gp170	107	0		hypothetical protein DRJ15_13150 [Bacteroidetes bacterium], RLD77627.1	96/47, 7e-23
ORF171	103 879	104 352	F	gp171	157	0	Deoxycytidylate deaminase, hydrolase	dCMP deaminase [Endozoicomonas ascidiicola], WP_067517971.1	87/46, 1e-33
ORF172	104 349	104 555	F	gp172	68	0		-	-
ORF173	104 555	104 866	F	gp173	103	0	Tail fiber protein	tail fiber protein [Klebsiella phage Metamorpho], QPB08810.1	69/43, 1e-6
ORF174	104 868	105 284	F	gp174	138	0		-	-
ORF175	105 285	105 452	F	gp175	55	0		-	-
ORF176	105 430	105 789	F	gp176	119	0	Head assembly cochaperone	putative head assembly cochaperone with GroEL [Cronobacter phage S13], YP_009196422.1	88/35, 4e-12
ORF177	105 918	106 211	F	gp177	97	0		-	-
ORF178	106 302	106 622	F	gp178	106	0		TPA: hypothetical protein [Cytophagales bacterium], HCX24606.1	65/47, 4e-12
ORF179	106 626	106 814	F	gp179	62	0		-	-
ORF180	106 994	107 596	R	gp180	200	0	Endonuclease	homing endonuclease HNH [Pseudoalteromonas phage SL20], YP_009791452.1	95/45, 1e-42
ORF181	107 687	107 824	F	gp181	45	0		hypothetical protein Ah1_00348 [Aeromonas phage Ah1], AUE22866.1	71/76, 1e-7

ORF182	107 821	108 261	F	gp182	146	0		-	-
ORF183	108 274	108 723	F	gp183	149	0		-	-
ORF184	108 738	109 055	F	gp184	105	0		-	-
ORF185	109 070	109 903	F	gp185	277	0	Thymidylate synthase	thymidylate synthase [Algibacillus agarilyticus], WP_111977819.1	99/78, 2e-165
ORF186	109 979	110 311	F	gp186	110	0		-	-
ORF187	110 314	111 024	F	gp187	236	0	Phosphotase, hydrolase	hypothetical protein G2285_00206 [Escherichia phage vB_EcoM_G2285], QBO62632.1	68/49, 1e-39
ORF188	111 017	111 262	F	gp188	81	0		-	-
ORF189	111 262	111 651	F	gp189	129	0		hypothetical protein [Photobacterium sp. BZF1], WP_187464313.1	90/48, 1e-29
ORF190	111 648	112 259	F	gp190	203	0		TPA: hypothetical protein [Gammaproteobacteria bacterium], HEB94359.1	45/52, 7e-18
ORF191	112 272	113 387	F	gp191	371	0	RNA ligase	hypothetical protein [Pseudoalteromonas phage J2-1], ATN93418.1	95/36, 3e-50
ORF192	113 384	113 695	F	gp192	103	1	Phosphotase, hydrolase	hypothetical protein GOVbin4162_64 [Prokaryotic dsDNA virus sp.], QDP59490.1	96/49, 1e-26
ORF193	113 692	113 940	F	gp193	82	0		-	-
ORF194	114 305	114 730	F	gp194	141	0	Cysteine dioxygenase	hypothetical protein DRJ15_12620 [Bacteroidetes bacterium], RLD77902.1	84/48, 3e-30
ORF195	114 799	114 960	F	gp195	53	0		-	-
ORF196	115 262	115 576	F	gp196	104	0		-	-
ORF197	115 576	115 977	F	gp197	133	0		-	-
ORF198	115 977	116 357	F	gp198	126	0		hypothetical protein [Acidobacteria bacterium], MBF84500.1	98/30, 1e-11
ORF199	116 354	116 749	F	gp199	131	0		hypothetical protein [Acidobacteria bacterium], MBF84500.1	94/34, 4e-12
ORF200	116 830	117 183	F	gp200	117	0		hypothetical protein [archaeon], NCQ52016.1	98/57, 5e-35
ORF201	117 281	117 448	F	gp201	55	0		-	-
ORF202	117 511	117 948	F	gp202	145	0		-	-

ORF203	117 966	118 841	F	gp203	291	0	Polynucleotide kinase/phosphatase	putative polynucleotide 5'-kinase and 3'-phosphatase [Cronobacter phage S13], YP_009196416.1	98/43, 6e-68
ORF204	119 192	119 653	F	gp204	153	0		-	-
ORF205	119 641	120 027	F	gp205	128	0		-	-
ORF206	120 029	120 328	F	gp206	99	0		-	-
ORF207	120 337	120 606	F	gp207	89	0		-	-
ORF208	120 621	120 944	F	gp208	107	0		TPA: hypothetical protein [Pseudoalteromonas sp.], HCV03393.1	93/55, 7e-31
ORF209	120 945	121 520	R	gp209	191	0	Baseplate wedge protein	gp3 tail completion and sheath stabilizer [Aeromonas phage PX29], YP_009011609.1	97/31, 5e-19
ORF210	121 517	122 986	R	gp210	489	0		-	-
ORF211	122 976	123 149	R	gp211	57	0	Baseplate hub assembly catalyst	hypothetical protein [Euryarchaeota archaeon], MBJ23124.1	96/43, 3e-6
ORF212	123 146	123 844	R	gp212	232	0	Baseplate protein	hypothetical protein [Candidatus Pacearchaeota archaeon], MAG26782.1	98/31, 1e-29
ORF213	123 866	124 531	R	gp213	221	0	Baseplate wedge protein	unnamed protein product [Aeromonas phage phiAS5], YP_003969616.1	76/36, 8e-24
ORF214	124 518	125 333	R	gp214	271	0	Endonuclease	hypothetical protein [Campylobacter coli], EAK2897022.1	73/38, 2e-14
ORF215	125 330	125 914	R	gp215	194	0	DNA end protector protein	DNA end protector protein [Shewanella phage Thanatos-2], QLA10743.1	93/48, 3e-50
ORF216	125 914	126 375	R	gp216	153	0	Head completion protein	decorative head protein [Serratia phage Muldoon], YP_009883869.1	93/57, 3e-50
ORF217	126 419	126 793	R	gp217	124	1		-	-
ORF218	126 793	127 065	R	gp218	90	1		-	-
ORF219	127 065	127 373	R	gp219	102	1		-	-
ORF220	127 387	127 767	R	gp220	126	0		-	-
ORF221	127 823	128 131	R	gp221	102	0		-	-
ORF222	128 128	128 322	R	gp222	64	0		-	-
ORF223	128 386	128 814	R	gp223	142	0		-	-
ORF224	128 814	129 029	R	gp224	71	0		-	-
ORF225	129 022	129 321	R	gp225	99	1		-	-

ORF226	129 308	129 592	R	gp226	94	0		-	-
ORF227	129 592	129 963	R	gp227	123	0		-	-
ORF228	129 967	131 409	R	gp228	480	0	DNA helicase	DNA helicase [Crocinitomicaceae bacterium], MBD78301.1	94/43, 2e-120
ORF229	131 500	132 495	F	gp229	331	0	Baseplate wedge subunit	baseplate tail tube cap [Aeromonas phage AS-szw], ATI17397.1	90/30, 1e-31
ORF230	132 492	133 055	F	gp230	187	0	Baseplate wedge subunit	baseplate wedge subunit [Aeromonas phage Ahp1_CNik-2021], QIW87125.1	97/35, 1e-25
ORF231	133 039	134 253	F	gp231	404	0	Baseplate protein	hypothetical protein [Acidobacteria bacterium], MBF84640.1	98/24, 7e-21
ORF232	134 255	135 217	F	gp232	320	0	Baseplate hub subunit and tail lysozyme	hypothetical protein [Acidobacteria bacterium], MBF84639.1	89/45, 3e-72
ORF233	135 232	135 597	F	gp233	121	0	Baseplate wedge subunit and lysozyme	gp25 baseplate wedge subunit [Aeromonas phage PX29], YP_009011622.1	90/41, 1e-21
ORF234	135 611	137 458	F	gp234	615	0	Baseplate wedge subunit	baseplate wedge subunit [Shigella phage SP18], YP_003934789.1	98/39, 9e-111
ORF235	137 458	143 505	F	gp235	2015	0	Baseplate wedge subunit	baseplate wedge initiator [Pseudomonas phage PspYZU05], ASD52067.1	51/27, 8e-95
ORF236	143 537	144 514	F	gp236	325	0	Baseplate wedge subunit	baseplate wedge subunit [Vibrio phage KVP40], NP_899589.1	99/41, 2e-74
ORF237	144 525	145 136	F	gp237	203	0		-	-
ORF238	145 140	146 846	F	gp238	568	0	Baseplate wedge subunit and tail pin	baseplate wedge subunit [Aeromonas phage phiAS4], YP_003969144.1	99/34, 2e-88
ORF239	146 843	147 586	F	gp239	247	0		-	-
ORF240	147 602	148 492	F	gp240	296	0	Neck protein (adaptor)	neck protein [Escherichia phage JSE], YP_002922224.1	96/41, 7e-67
ORF241	148 499	149 152	F	gp241	217	0	DNA binding protein	TPA: hypothetical protein [Methylococcaceae bacterium], HHZ71316.1	96/43, 5e-51
ORF242	149 139	149 891	F	gp242	250	0	Neck protein (head closure)	neck protein [Vibrio phage VH7D], YP_009006279.1	88/45, 6e-47
ORF243	149 903	150 724	F	gp243	273	0	Tail sheath stabilizer and completion protein	gp15 tail sheath stabilizer and completion protein [Enterobacteria phage RB16], YP_003858504.1	83/44, 1e-50

a. F, forward; R, reverse.
b. TMHs, transmembrane helices, searched with TMHMM Server v. 2.0.
c. No significant similarity found.
