# Supplementary material for: Virus-Host Interactions and Genetic Diversity of Antarctic Sea Ice Bacteriophages
Source: mBio. 2022 May 9;13(3):e00651-22. doi: 10.1128/mbio.00651-22 (PMC9239159; doi:10.1128/mbio.00651-22)
Supplement: FIG S2 [file mbio.00651-22-s0007.pdf]

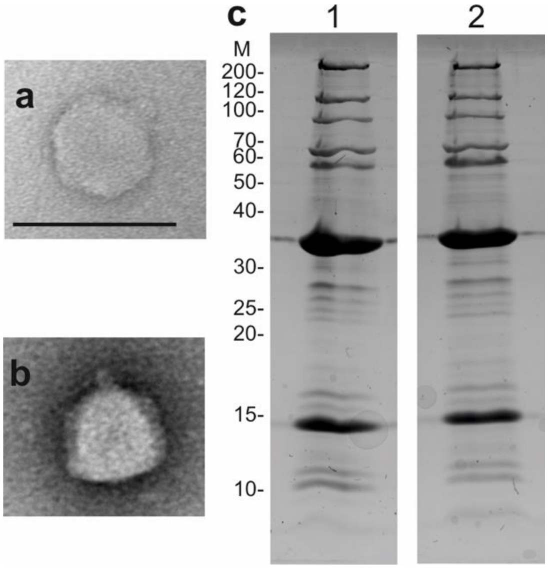

Figure S2. Transmission electron micrographs of OANV1 virus particles stained with (a) uranyl acetate (2%, w/v) or (b) Nano-W. Scale bar is 100 nm in a for a and b. (c) Polyacrylamide gel electrophoresis of purified virus samples which were prepared in parallel (labeled 1 and 2; 10 μg each) and used in TEM in a and b, respectively. M, marker (PageRuler unstained protein ladder).
