# Supplementary material for: Virus-Host Interactions and Genetic Diversity of Antarctic Sea Ice Bacteriophages
Source: mBio. 2022 May 9;13(3):e00651-22. doi: 10.1128/mbio.00651-22 (PMC9239159; doi:10.1128/mbio.00651-22)
Supplement: FIG S3 [file mbio.00651-22-s0008.pdf]

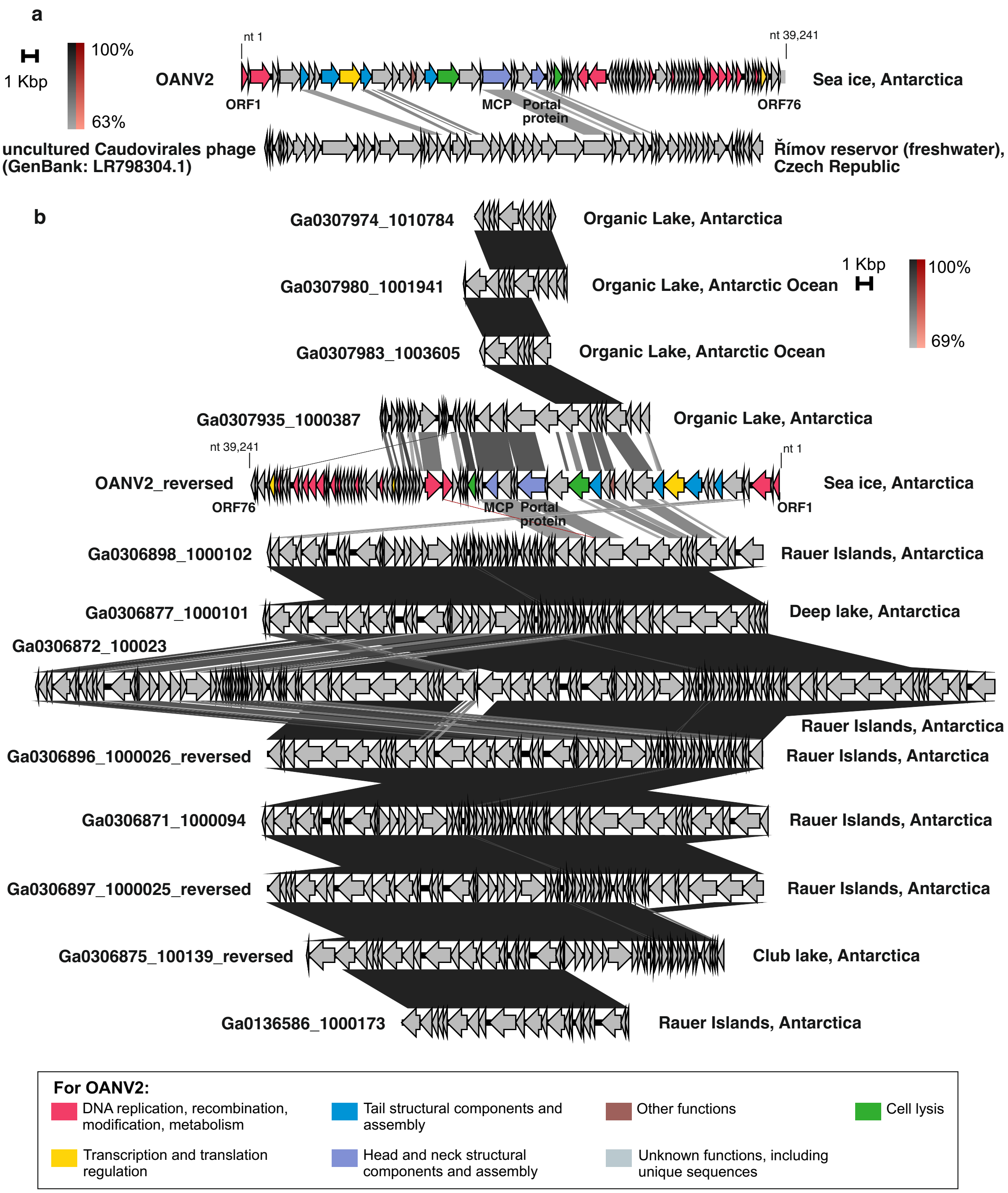

Figure S3. OANV2 and the selection of similar virus genome sequences: (a) uncultured Caudovirales phage (Genbank accession number LR798304.1) found with blastx search against nr protein database and (b) scaffolds found with blastn search against IMG/VR database. Full list of scaffolds is presented in Table S5. Here, those scaffolds that were identical to a part of some other scaffold are excluded. ORFs and genes are shown as arrows, and regions that are similar between sequences are shown as shadings (blastn, E-value threshold of 0.001, grey for direct and red for inverted similarities, from (a) 63 to 100% or (b) 69 to 100%). Note that OANV2 and some other sequences are reversed in (b). Color codes for OANV2 ORFs are shown in the lower panel. Sampling locations are marked on the right. The figure was generated using Easyfig v. 2.2.2.
