# Supplementary material for: Virus-Host Interactions and Genetic Diversity of Antarctic Sea Ice Bacteriophages
Source: mBio. 2022 May 9;13(3):e00651-22. doi: 10.1128/mbio.00651-22 (PMC9239159; doi:10.1128/mbio.00651-22)
Supplement: TABLE S2 [file mbio.00651-22-s0002.pdf]

Table S2. Putative functions assigned to PANV2 ORF products.

ORF	Start, nt	Stop, nt	Direction[a]	Gene product (gp)	Protein size, aa	TMHs[b]	Putative function	**Best Blastx match** (thresholds: E-value 1e-5, query cover 30%, identity 30%, search dated 18.02.2021)	
								**Protein [organism], accession number**	**Query cover/identity, %, E-value**
ORF1	1	585	F	gp1	194	0	Terminase small subunit	TPA: terminase small subunit [Methylococcaceae bacterium], HHZ70429.1	75/72, 6e-72
ORF2	572	1 828	F	gp2	418	0	Terminase large subunit	TPA: terminase [Glaciecola sp.], HAQ49901.1	97/72, 0
ORF3	1 839	3 281	F	gp3	480	0	Portal protein	Putative prophage PSSB64-02 [uncultured Mediterranean phage uvMED], BAQ84211.1	95/50, 7e-157
ORF4	3 281	4 327	F	gp4	348	0	Head morphogenesis protein	minor capsid protein [Pantoea septica], WP_084885774.1	97/40, 2e-64
ORF5	4 380	5 033	F	gp5	217	0		Putative prophage PSSB64-02 [uncultured Mediterranean phage uvMED], BAQ84212.1	88/47, 3e-24
ORF6	5 047	6 204	F	gp6	385	2	Major capsid protein	TPA: P22 coat - protein 5 family protein [Shewanella frigidimarina], HBF47038.1	99/69, 9e-173
ORF7	6 252	6 446	R	gp7	64	0		-[c]	-
ORF8	6 443	6 934	R	gp8	163	0		hypothetical protein COA78_21100 [Blastopirellula sp.], PHS02420.1	62/39, 4e-13
ORF9	6 927	7 112	R	gp9	61	0		-	-
ORF10	7 109	7 411	R	gp10	100	0		-	-
ORF11	7 411	7 638	R	gp11	75	0		-	-
ORF12	7 626	7 865	R	gp12	79	0		-	-
ORF13	7 987	8 118	F	gp13	43	0		-	-
ORF14	8 115	8 729	F	gp14	204	0		-	-
ORF15	8 722	9 114	F	gp15	130	0		-	-
ORF16	9 124	9 924	F	gp16	266	0		TPA: hypothetical protein [Flavobacteriales bacterium], HHZ97169.1	88/51, 4e-61
ORF17	9 924	10 289	F	gp17	121	0		TPA: hypothetical protein [Methylococcaceae bacterium], HHZ69969.1	99/48, 3e-17

ORF18	10 289	10 750	F	gp18	153	0	Tail protein	TPA: hypothetical protein [Flavobacteriales bacterium], HHZ97787.1	99/57, 3e-53
ORF19	10 750	11 169	F	gp19	139	0	Tail protein	TPA: hypothetical protein [Flavobacteriales bacterium], HHZ94507.1	84/49, 2e-33
ORF20	11 184	11 597	F	gp20	137	0	Portal/tail protein	-	-
ORF21	11 599	12 042	F	gp21	147	0		hypothetical protein [Alteromonas sp.], MBU35306.1	88/38, 1e-23
ORF22	12 148	14 094	F	gp22	648	0	Tail tape measure protein	TPA: hypothetical protein [Methylococcaceae bacterium], HHZ68955.1	40/57, 6e-73
ORF23	14 091	14 819	F	gp23	242	1		hypothetical protein [Alteromonas sp.], MBU35304.1	99/52, 4e-72
ORF24	14 816	17 113	F	gp24	765	0	Tail fiber protein	hypothetical protein [Alteromonas sp.], MBU35303.1	84/58, 0
ORF25	17 124	17 375	F	gp25	83	0		-	-
ORF26	17 375	19 447	F	gp26	690	0		-	-
ORF27	19 484	19 627	F	gp27	47	0		-	-
ORF28	19 624	20 097	F	gp28	157	4		-	-
ORF29	20 094	20 372	F	gp29	92	2		-	-
ORF30	20 369	20 710	F	gp30	113	0	Transpeptidase	hypothetical protein Tp138OMZ00d2C19078241_28 [Prokaryotic dsDNA virus sp.], QDP55696.1	69/51, 3e-17
ORF31	20 710	21 090	F	gp31	126	2		hypothetical protein [Pseudoalteromonas sp.], NRA79365.1	99/65, 2e-45
ORF32	21 091	21 219	R	gp32	42	1		-	-
ORF33	21 209	21 346	R	gp33	45	1		-	-
ORF34	21 339	21 488	R	gp34	49	0		-	-
ORF35	21 485	21 652	R	gp35	55	0	RNA polymerase sigma factor	-	-
ORF36	21 649	21 900	R	gp36	83	0	Transcriptional regulator	-	-
ORF37	21 884	22 165	R	gp37	93	0		-	-
ORF38	22 190	22 507	R	gp38	105	0		hypothetical protein B1M_00505 [Burkholderia sp. TJI49], EGD06613.1	36/58, 6e-6
ORF39	22 762	22 965	R	gp39	67	1		-	-

ORF40	22 968	23 177	R	gp40	69	0		hypothetical protein [Parvibaculum sp.], MAN63724.1	80/54, 2e-12
ORF41	23 189	23 473	R	gp41	94	3		-	-
ORF42	23 475	23 765	R	gp42	96	0	Nuclease/hydrolase	TPA: hypothetical protein [Flavobacteriales bacterium], HHZ94256.1	94/73, 2e-38
ORF43	23 766	24 281	R	gp43	171	0	Single-strand DNA-binding protein	single-strand DNA-binding protein [Paraglaciecola arctica], WP_007618529.1	65/91, 1e-69
ORF44	24 294	24 992	R	gp44	232	0		hypothetical protein [Chloroflexi bacterium], MBG7617472.1	87/53, 3e-52
ORF45	24 989	25 531	R	gp45	180	0	Single-strand DNA-binding protein	single-strand DNA binding protein [Pseudoalteromonas phage Pq0], YP_009226084.1	77/76, 5e-73
ORF46	25 524	25 661	R	gp46	45	0		-	-
ORF47	25 795	26 001	F	gp47	68	0		-	-
ORF48	26 001	26 261	F	gp48	86	0		-	-
ORF49	26 261	26 707	F	gp49	148	0	Single-strand DNA-binding protein	hypothetical protein [Pseudoalteromonas sp.], MAE02312.1	73/43, 5e-17
ORF50	26 708	26 905	F	gp50	65	0		hypothetical protein [Pseudoalteromonas virus vB_PspS-H6/1], ANJ65543.1	92/55, 1e-11
ORF51	26 902	27 297	F	gp51	131	0	Nuclease/hydrolase	hypothetical protein [Pseudomonadales bacterium], MBA57003.1	99/54, 3e-45
ORF52	27 433	27 624	F	gp52	63	0		-	-
ORF53	27 612	27 821	F	gp53	69	0		-	-
ORF54	27 814	28 005	F	gp54	63	0		-	-
ORF55	28 243	28 710	F	gp55	155	0	Phage regulatory protein Rha	Antirepressor protein [Prokaryotic dsDNA virus sp.], QDP50490.1	98/60, 2e-63
ORF56	28 721	29 443	F	gp56	240	0		hypothetical protein [Pseudoalteromonas phage Pq0], YP_009226075.1	99/55, 1e-80
ORF57	29 447	30 769	F	gp57	440	0	Replicative DNA helicase	replicative DNA helicase [Leucothrix sargassi], RVU85441.1	97/33, 7e-65
ORF58	30 773	31 030	F	gp58	85	0		-	-
ORF59	31 030	31 263	F	gp59	77	0		-	-
ORF60	31 330	31 488	F	gp60	52	0		-	-

ORF61	31 680	31 856	F	gp61	58	0	RNA-binding protein	hypothetical protein NVP1023O_68 [Vibrio phage 1.023.O._10N.222.51.B4], AUR82377.1	96/56, 1e-12
ORF62	31 873	32 016	F	gp62	47	1		-	-
ORF63	32 018	32 161	F	gp63	47	0		-	-
ORF64	32 158	32 391	F	gp64	77	0		hypothetical protein [Aeromonas salmonicida], WP_044303429.1	98/42, 2e-10
ORF65	32 388	32 585	F	gp65	65	0		-	-
ORF66	32 585	32 800	F	gp66	71	0		hypothetical protein [Parashewanella curva], WP_165904988.1	68/63, 6e-10
ORF67	32 818	33 006	F	gp67	62	0		-	-
ORF68	33 003	33 410	F	gp68	135	0		-	-
ORF69	33 407	33 577	F	gp69	56	0		-	-
ORF70	33 658	34 410	F	gp70	250	0		-	-
ORF71	34 413	34 850	F	gp71	145	0		-	-
ORF72	34 847	35 227	F	gp72	126	0		hypothetical protein [Colwellia sp.], MBL4941164.1	51/57, 2e-12
ORF73	35 231	35 683	F	gp73	150	0		TPA: hypothetical protein [Glaciecola sp.], HAQ49903.1	99/64, 1e-59

a. F, forward; R, reverse.
b. TMHs, transmembrane helices, searched with TMHMM Server v. 2.0.
c. No significant similarity found.
