# Supplementary material for: Virus-Host Interactions and Genetic Diversity of Antarctic Sea Ice Bacteriophages
Source: mBio. 2022 May 9;13(3):e00651-22. doi: 10.1128/mbio.00651-22 (PMC9239159; doi:10.1128/mbio.00651-22)
Supplement: TABLE S3 [file mbio.00651-22-s0003.pdf]

Table S3. Putative functions assigned to OANV1 ORF products.

ORF	Start, nt	Stop, nt	Direction[a]	Gene product (gp)	Protein size, aa	TMHs[b]	Putative function	**Best Blastx match** (thresholds: E-value 1e-5, query cover 30%, identity 30%, search dated 18.02.2021)	
								**Protein [organism], accession number**	**Query cover/identity, %, E-value**
ORF1	1	639	F	gp1	212	0	Terminase small subunit	terminase small subunit [Xanthobacter sp. 29k], WP_149576359.1	75/36, 1e-9
ORF2	737	961	F	gp2	74	0		-[c]	-
ORF3	965	2 479	F	gp3	504	0	Terminase large subunit	terminase [Sutterellaceae bacterium], MAG80870.1	93/57, 0
ORF4	2 476	2 763	F	gp4	95	0		-	-
ORF5	2 768	4 477	F	gp5	569	0	Portal protein	hypothetical protein [Planctomycetes bacterium], NBC11671.1	98/52, 0
ORF6	4 509	4 823	F	gp6	104	0		-	-
ORF7	4 813	5 619	F	gp7	268	0	Protease	hypothetical protein [Pleomorphomonas oryzae], WP_051229201.1	48/39, 1e-14
ORF8	5 625	6 623	F	gp8	332	0	Major capsid protein	phage capsid protein [Rhizobium oryzihabitans], WP_164056307.1	99/64, 6e-147
ORF9	6 695	7 126	F	gp9	143	0	Major capsid protein	hypothetical protein [Rhizobium sp. RM], WP_138792359.1	97/54, 1e-41
ORF10	7 130	7 573	F	gp10	147	0		-	-
ORF11	7 653	8 300	F	gp11	215	0	Tail tubular protein	hypothetical protein [Terrimicrobium sacchariphilum], WP_075079046.1	78/37, 3e-19
ORF12	8 304	10 643	F	gp12	779	0	Portal protein	hypothetical protein [Agaricicola taiwanensis], WP_188411061.1	99/35, 4e-130
ORF13	10 640	11 098	F	gp13	152	0	Acetyltransferase	hypothetical protein B7Y08_14015 [Rhodospirillales bacterium 24-66-33], OYZ94247.1	85/37, 7e-15
ORF14	11 100	11 603	F	gp14	167	0		hypothetical protein [Agrobacterium sp. AGB01], WP_192491282.1	98/37, 5e-21
ORF15	11 616	14 465	F	gp15	949	0	Lysozyme	-	-
ORF16	14 485	23 445	F	gp16	2986	0	Lysin containing a β/γ crystallin motif	hypothetical protein E5V54_11180 [Mesorhizobium sp.], TIW56675.1	63/47, 0
ORF17	23 448	24 116	F	gp17	222	0	Tailspike protein	-	-

ORF18	24 135	24 533	F	gp18	132	0		hypothetical protein [Leisingera sp. ANG-M7], WP_039178778.1	93/74, 1e-59
ORF19	24 549	24 857	F	gp19	102	0		-	-
ORF20	24 868	26 430	F	gp20	520	0		-	-
ORF21	26 432	26 911	F	gp21	159	0		hypothetical protein [Rhizobium album], WP_109458139.1	88/46, 6e-23
ORF22	26 908	27 645	F	gp22	245	0		hypothetical protein [Blastomonas sp. CCH2-A2], WP_156456616.1	86/32, 4e-10
ORF23	27 655	27 978	F	gp23	107	3		-	-
ORF24	27 965	28 120	F	gp24	51	1		-	-
ORF25	28 120	28 785	F	gp25	221	0	Lysozyme	hypothetical protein SUFG_00006 [Sulfitobacter phage phiCB2047-B], YP_007675795.1	97/64, 4e-90
ORF26	28 889	29 215	F	gp26	108	1		hypothetical protein HYP63_gp65 [Ruegeria phage vB_RpoP-V13], YP_009880553.1	94/34, 4e-7
ORF27	29 115	29 432	F	gp27	105	0		hypothetical protein EE36P1_gp67 [Sulfitobacter phage EE36phi1], YP_002898998.1	71/52, 7e-11
ORF28	29 404	29 649	R	gp28	81	0		-	-
ORF29	29 651	30 709	R	gp29	352	0	DNA polymerase	DNA-directed DNA-polymerase, family A, palm domain containing protein [uncultured Caudovirales phage], CAB4157539.1	92/37, 2e-45
ORF30	30 706	30 960	R	gp30	84	0		-	-
ORF31	30 960	31 169	R	gp31	69	2		-	-
ORF32	31 169	32 425	R	gp32	418	0	DNA polymerase	hypothetical protein EHM67_00245 [Hyphomicrobiaceae bacterium], RPI47093.1	92/33, 1e-38
ORF33	32 422	32 586	R	gp33	54	0		-	-
ORF34	32 649	33 488	R	gp34	279	0		hypothetical protein UFOVP893_28 [uncultured Caudovirales phage], CAB4168979.1	63/37, 2e-28
ORF35	33 514	35 082	R	gp35	522	0	Helicase/nuclease	protein of unknown function DUF2800 [uncultured Caudovirales phage], CAB4242121.1	84/34, 4e-43
ORF36	35 075	35 227	R	gp36	50	0		-	-

ORF37	35 226	35 561	F	gp37	111	0	Nuclease	VRR-NUC domain-containing protein [Facklamia hominis], WP_006907999.1	74/45, 1e-15
ORF38	35 609	35 836	F	gp38	75	0		-	-
ORF39	35 836	37 281	F	gp39	481	0	DNA helicase	hypothetical protein [Planctomycetes bacterium], NBC11663.1	98/41, 1e-98
ORF40	37 278	37 592	F	gp40	104	0		-	-
ORF41	37 652	40 129	F	gp41	825	0	DNA primase/polymerase	bifunctional DNA primase/polymerase [Shinella sp. DD12], EYR80005.1	93/32, 2e-93
ORF42	40 198	40 599	R	gp42	133	0		-	-
ORF43	40 599	40 874	R	gp43	91	0		-	-
ORF44	40 878	41 180	R	gp44	100	0		hypothetical protein [Gemmobacter caeni], WP_108129344.1	74/56, 6e-17
ORF45	41 177	41 704	R	gp45	175	1		-	-
ORF46	41 704	42 033	R	gp46	109	0		-	-
ORF47	42 140	42 583	R	gp47	147	0		-	-
ORF48	42 637	42 957	R	gp48	106	0		-	-
ORF49	42 976	43 299	R	gp49	107	0	Endoribonuclease	-	-
ORF50	43 516	43 833	R	gp50	105	0		-	-
ORF51	43 830	44 588	R	gp51	252	0		-	-
ORF52	44 585	44 953	R	gp52	122	0		-	-
ORF53	44 938	45 165	R	gp53	75	0		-	-
ORF54	45 162	45 380	R	gp54	72	0		Lar family restriction alleviation protein [Leisingera sp. ANG-M7], WP_039178906.1	94/55, 1e-18
ORF55	45 377	45 649	R	gp55	90	0		-	-
ORF56	45 646	46 119	R	gp56	157	0		-	-
ORF57	46 119	46 286	R	gp57	55	0		-	-
ORF58	46 286	46 456	R	gp58	56	0		-	-
ORF59	46 527	46 817	R	gp59	96	0		-	-
ORF60	46 889	47 281	R	gp60	130	0		-	-
ORF61	47 356	47 739	R	gp61	127	0		hypothetical protein HWB26_gp02 [Lentibacter virus vB_LenP_ICBM2], YP_009834189.1	31/70, 1e-9

a. F, forward; R, reverse.
b. TMHs, transmembrane helices, searched with TMHMM Server v. 2.0.
c. No significant similarity found.
