# Supplementary material for: Virus-Host Interactions and Genetic Diversity of Antarctic Sea Ice Bacteriophages
Source: mBio. 2022 May 9;13(3):e00651-22. doi: 10.1128/mbio.00651-22 (PMC9239159; doi:10.1128/mbio.00651-22)
Supplement: TABLE S4 [file mbio.00651-22-s0004.pdf]

Table S4. Putative functions assigned to OANV2 ORF products.

ORF	Start, nt	Stop, nt	Direction[a]	Gene product (gp)	Protein size, aa	TMHs[b]	Putative function	**Best Blastx match** (thresholds: E-value 1e-5, query cover 30%, identity 30%, search dated 18.02.2021)	
								**Protein [organism], accession number**	**Query cover/identity, %, E-value**
ORF1	1	450	F	gp1	149	0	Terminase small subunit	phage terminase small subunit [Cellulophaga phage phi19:1], YP_008241758.1	81/51, 4e-32
ORF2	461	628	F	gp2	55	0		-[c]	-
ORF3	625	2 085	F	gp3	486	0	Terminase large subunit	hypothetical protein [Paracoccus sulfuroxidans], WP_199756571.1	99/74, 0
ORF4	2 238	2 606	F	gp4	122	0		-	-
ORF5	2 606	2 752	F	gp5	48	0		-	-
ORF6	2 752	4 233	F	gp6	493	0		hypothetical protein [Alteromonadaceae bacterium], MAL99405.1	61/44, 7e-55
ORF7	4 230	4 850	F	gp7	206	0	Tail tubular protein	hypothetical protein UFOVP373_19 [uncultured Caudovirales phage], CAB5222614.1	99/64, 2e-97
ORF8	4 852	5 232	F	gp8	126	0		hypothetical protein UFOVP373_20 [uncultured Caudovirales phage], CAB5222622.1	98/65, 6e-51
ORF9	5 273	5 635	F	gp9	120	0		hypothetical protein E4H01_10375 [Xanthomonadales bacterium], TFH46180.1	96/70, 3e-51
ORF10	5 753	7 060	F	gp10	435	0	Tailspike protein	-	-
ORF11	7 065	8 591	F	gp11	508	0	Ribonuclease E	hypothetical protein [Porticoccaceae bacterium], MBL4781782.1	40/40, 3e-40
ORF12	8 592	9 398	F	gp12	268	0	Tail fiber protein	MdpB Microcystin-dependent protein [uncultured Caudovirales phage], CAB5222652.1	60/59, 9e-35
ORF13	9 410	10 888	F	gp13	492	0		TPA: hypothetical protein [Flavobacteriales bacterium], HHZ96248.1	99/61, 0
ORF14	10 885	11 376	F	gp14	163	0		hypothetical protein UFOVP1469_33 [uncultured Caudovirales phage], CAB4214963.1	48/48, 1e-10
ORF15	11 397	12 227	F	gp15	276	0		hypothetical protein [Rhizobiales bacterium], MBI2718149.1	91/35, 4e-25

ORF16	12 224	12 580	F	gp16	118	0	Acetyltransferase	putative acetyltransferase [uncultured Mediterranean phage uvMED], BAQ89700.1	96/49, 9e-36
ORF17	12 577	13 230	F	gp17	217	0		hypothetical protein Unbinned1502contig1001_24 [Prokaryotic dsDNA virus sp.], QDP54037.1	56/50, 4e-28
ORF18	13 231	14 136	F	gp18	301	0	Tail spike protein/chaperone	hypothetical protein [Phycisphaerae bacterium], MAO23898.1	82/40, 2e-19
ORF19	14 136	15 707	F	gp19	523	0	Cell wall hydrolase	hypothetical protein [Rhodobacteraceae bacterium R_SAG2], NKX42100.1	52/38, 1e-37
ORF20	15 707	17 218	F	gp20	503	0		hypothetical protein [Roseovarius nubinhibens], WP_009813644.1	90/47, 2e-107
ORF21	17 223	17 402	F	gp21	59	0		-	-
ORF22	17 393	19 471	F	gp22	692	0	Portal protein	hypothetical protein UFOVP373_33 [uncultured Caudovirales phage], CAB5222808.1	98/62, 0
ORF23	19 468	19 689	F	gp23	73	0		-	-
ORF24	19 682	19 927	F	gp24	81	0		-	-
ORF25	19 914	20 903	F	gp25	329	0		hypothetical protein UFOVP373_35 [uncultured Caudovirales phage], CAB5222832.1	73/46, 2e-57
ORF26	20 925	21 881	F	gp26	318	0	Major capsid protein	Family of unknown function (DUF5309) [uncultured Caudovirales phage], CAB5222848.1	99/73, 1e-162
ORF27	22 058	22 363	F	gp27	101	0		hypothetical protein UFOVP373_38 [uncultured Caudovirales phage], CAB5222867.1	95/62, 1e-33
ORF28	22 350	22 565	F	gp28	71	2		-	-
ORF29	22 562	23 155	F	gp29	197	0	Glycosyl hydrolase/lysozyme	carboxypeptidase [Lentilitoribacter sp. Alg239-R112], WP_162651541.1	88/69, 4e-83
ORF30	23 152	23 292	F	gp30	46	0		-	-
ORF31	23 289	23 471	F	gp31	60	2		-	-
ORF32	23 468	23 713	F	gp32	81	1		-	-
ORF33	23 697	23 852	F	gp33	51	0		-	-
ORF34	23 860	24 255	R	gp34	131	1		hypothetical protein [Methylobacterium sp. 190mf], WP_103984825.1	57/40, 5e-7
ORF35	24 252	24 989	R	gp35	245	0	DNA-methylase	hypothetical protein COA96_10290 [SAR86 cluster bacterium], PCJ24125.1	99/46, 6e-61

ORF36	25 059	26 294	R	gp36	411	0	DNA polymerase subunit	hypothetical protein E5W06_00175 [Mesorhizobium sp.], TIU88819.1	99/48, 2e-122
ORF37	26 433	26 735	R	gp37	100	0		hypothetical protein [Rhodobacteraceae bacterium], NBT30483.1	83/64, 3e-30
ORF38	26 732	27 001	R	gp38	89	0		-	-
ORF39	27 003	27 203	R	gp39	66	0		-	-
ORF40	27 246	27 458	R	gp40	70	1		-	-
ORF41	27 469	27 723	R	gp41	84	0		-	-
ORF42	27 720	27 914	R	gp42	64	0		-	-
ORF43	27 925	28 212	R	gp43	95	0		-	-
ORF44	28 308	28 541	F	gp44	77	0		hypothetical protein EOS70_27915 [Mesorhizobium sp.], RWC28140.1	94/51, 1e-16
ORF45	28 553	28 759	F	gp45	68	0	DNA binding protein	hypothetical protein [Sphingomonas gellani], WP_093663927.1	85/46, 6e-9
ORF46	28 756	29 127	F	gp46	123	0		-	-
ORF47	29 127	29 453	F	gp47	108	0		-	-
ORF48	29 446	29 730	F	gp48	94	0	Nuclease/hydrolyse	nuclease [Sulfitobacter phage phiGT1], QLB38267.1	88/51, 5e-21
ORF49	29 886	30 698	F	gp49	270	0		hypothetical protein COA96_10295 [SAR86 cluster bacterium], PCJ24126.1	84/49, 4e-48
ORF50	30 685	31 047	F	gp50	120	0		hypothetical protein [Phaeobacter sp. S60], WP_040182831.1	88/64, 9e-40
ORF51	31 044	31 247	F	gp51	67	0		-	-
ORF52	31 244	31 564	F	gp52	106	0	Replication initiator protein	hypothetical protein BEN50_21540 [Euhalothece sp. KZN 001], PNW26769.1	65/47, 1e-8
ORF53	31 561	31 779	F	gp53	72	0		hypothetical protein [Salipaludibacillus aurantiacus], WP_093050966.1	73/52, 1e-12
ORF54	31 792	32 037	F	gp54	81	0		-	-
ORF55	32 049	32 312	F	gp55	87	0		-	-
ORF56	32 309	32 578	F	gp56	89	0		-	-
ORF57	32 581	32 796	F	gp57	71	0	DNA binding protein	putative transcription factor [Sulfitobacter phage phiGT1], QLB38251.1	91/68, 1e-22
ORF58	32 793	32 975	F	gp58	60	2		hypothetical protein [Salmonella enterica], EGH2615417.1	95/47, 2e-6
ORF59	32 975	33 355	F	gp59	126	0	Single-stranded DNA binding protein	single-stranded DNA-binding protein [Paracoccus suum], WP_114076349.1	99/52, 3e-33
ORF60	33 463	33 612	F	gp60	49	0		-	-
ORF61	33 643	33 819	F	gp61	58	0		-	-

ORF62	33 816	34 409	F	gp62	197	0	Single strand-annealing protein	ERF family protein [Rhodobacter xinxiangensis], WP_136685662.1	97/60, 2e-74
ORF63	34 406	35 002	F	gp63	198	0	DNA methylase	hypothetical protein [Alteromonas phage PB15], APC46559.1	98/78, 1e-109
ORF64	34 999	35 418	F	gp64	139	0	DNA binding protein	hypothetical protein [Sulfitobacter sp. HI0054], WP_067266081.1	97/50, 1e-34
ORF65	35 446	35 718	F	gp65	90	0		-	-
ORF66	35 729	36 121	F	gp66	130	0	Recombination nuclease	DUF1364 family protein [Profundibacterium mesophilum], WP_159966149.1	69/64, 2e-35
ORF67	36 353	36 550	F	gp67	65	0		-	-
ORF68	36 573	36 893	F	gp68	106	0		-	-
ORF69	36 890	37 072	F	gp69	60	0		-	-
ORF70	37 069	37 305	F	gp70	78	0	DNA binding protein	hypothetical protein JT311_gp33 [Ruegeria phage vB_RpoS-V16], YP_009997167.1	83/41, 4e-10
ORF71	37 302	37 472	F	gp71	56	2		-	-
ORF72	37 485	37 886	F	gp72	133	0	Transcriptional regulator	hypothetical protein OA238_c06650 [Octadecabacter arcticus 238], AGI70889.1	95/50, 1e-28
ORF73	37 896	38 057	F	gp73	53	0		-	-
ORF74	38 238	38 762	F	gp74	174	0		-	-
ORF75	38 759	38 944	F	gp75	61	0		-	-
ORF76	38 941	8	F	gp76	102	0		hypothetical protein [Sphingomonas sp. YJ09], WP_155262969.1	83/57, 2e-25

a. F, forward; R, reverse.
b. TMHs, transmembrane helices, searched with TMHMM Server v. 2.0.
c. No significant similarity found.
