# Supplementary material for: Virus-Host Interactions and Genetic Diversity of Antarctic Sea Ice Bacteriophages
Source: mBio. 2022 May 9;13(3):e00651-22. doi: 10.1128/mbio.00651-22 (PMC9239159; doi:10.1128/mbio.00651-22)
Supplement: TABLE S5 [file mbio.00651-22-s0005.pdf]

**Table S5.** Scaffolds with several (at least three) regions recruited as hits in the blast search against IMG/VR with Antarctic virus isolates whole genomes as queries (dated 9.12.2020).

| Scaffold ID | Genome ID | Length, bp | Additional notes | Genome | Ecosystem | Reference[a] | Overall identity to query genome, % |
|---|---|---|---|---|---|---|---|
| **Hits to PANV2 (35 731 bp)** | | | | | | | |
| 3300009136 assembled Ga0118735_10000119 | 3300009136 | 22 712 | | Marine sediment microbial communities from methane seeps within Hudson Canyon, US Atlantic Margin - Hudson Canyon PC-16 82 cmbsf (*) (MER-FS) (assembled) | Environmental; Aquatic; Sediment | | 41.3 |
| 3300022822 assembled Ga0222646_100076 | 3300022822 | 36 531 | | Saline water microbial communities from Ace Lake, Antarctica - #293 (*) (MER-FS) (assembled) | Environmental; Aquatic; Non-marine Saline and Alkaline; Saline | (1) | 43.0 |
| 3300022841 assembled Ga0222644_1000416 | 3300022841 | 13 576 | | Saline water microbial communities from Ace Lake, Antarctica - #291 (*) (MER-FS) (assembled) | Environmental; Aquatic; Non-marine Saline and Alkaline; Saline | (1) | 25.8 |
| 3300022839 assembled Ga0222649_1001735 | 3300022839 | 5 400 | Identical to a part of Ga0222646_100076 | Saline water microbial communities from Ace Lake, Antarctica - #337 (*) (MER-FS) (assembled) | Environmental; Aquatic; Non-marine Saline and Alkaline; Saline | (1) | 11.0 |
| **Hits to OANV1 (48 354 bp)** | | | | | | | |
| JGI24025J20009_10000218 | 3300001749 | 44 623 | Later reassembled as Ga0209121_10000387 | Oil polluted marine microbial communities from Coal Oil Point, Santa Barbara, California, USA - Sample 3 (*) (MER-FS) (assembled) | Environmental; Aquatic; Marine; Oil seeps | (2, 3) | 46.9 |

| | | | | | | | |
|---|---|---|---|---|---|---|---|
| Ga0209121_10000387 | 3300027742 | 46 428 | | Oil polluted marine microbial communities from Coal Oil Point, Santa Barbara, California, USA - Sample 3 (SPAdes) (*) (MER-FS) (assembled) | Environmental; Aquatic; Marine; Oil seeps | (2, 3) | 46.7 |
| Ga0075467_10002611 | 3300006803 | 14 202 | Identical to a part of Ga0208643_1000827 | Aqueous microbial communities from the Delaware River and Bay under freshwater to marine salinity gradient to study organic matter cycling in a time-series - DEBay_Spr_20_>0.8_DNA (*) (MER-FS) (assembled)' | Environmental; Aquatic; Marine; Coastal | (4) | 21.9 |
| Ga0070748_1002588 | 3300006920 | 8 362 | | Aqueous microbial communities from the Delaware River and Bay under freshwater to marine salinity gradient to study organic matter cycling in a time-series - Viral MetaG DEL_Nov_12 (*) (MER-FS) (assembled) | Environmental; Aquatic; Marine; Coastal | (4) | 13.6 |
| Ga0208643_1000827 | 3300025645 | 19 117 | | Aqueous microbial communities from the Delaware River and Bay under freshwater to marine salinity gradient to study organic matter cycling in a time-series - Viral MetaG DEL_Nov_12 (SPAdes) (*) (MER-FS) (assembled) | Environmental; Aquatic; Marine; Coastal | (4) | 27.9 |
| **Hits to OANV2 (39 241 bp)** | | | | | | | |
| Ga0307971_1001547 | 3300031382 | 11 581 | Identical to a part of Ga0307935_1000387 | Saline water microbial communities from Organic Lake, Antarctica - #714 (*) (MER-FS) (assembled) | Environmental; Aquatic; Non-marine Saline | (5) | 24.1 |

| | | | | | | | |
|---|---|---|---|---|---|---|---|
| | | | | | and Alkaline; Saline | | |
| Ga0307983_1003605 | 3300031269 | 5 263 | | Saline water microbial communities from Organic Lake, Antarctica - #991 (*) (MER-FS) (assembled) | Environmental; Aquatic; Non-marine Saline and Alkaline; Saline | (5) | 10.7 |
| Ga0307980_1001941 | 3300031216 | 7 701 | | Saline water microbial communities from Organic Lake, Antarctica - #1060 (*) (MER-FS) (assembled) | Environmental; Aquatic; Non-marine Saline and Alkaline; Saline | (5) | 15.0 |
| Ga0307935_1000387 | 3300031215 | 20 059 | | Saline water microbial communities from Organic Lake, Antarctica - #92 (*) (MER-FS) (assembled) | Environmental; Aquatic; Non-marine Saline and Alkaline; Saline | (5) | 33.2 |
| Ga0307974_1010784 | 3300031211 | 6 026 | | Saline water microbial communities from Organic Lake, Antarctica - #784 (*) (MER-FS) (assembled) | Environmental; Aquatic; Non-marine Saline and Alkaline; Saline | (5) | 12.1 |
| Ga0306875_100139 | 3300028402 | 31 011 | | Saline lake microbial communities from Club lake, Antarctica - Metagenome #318 (v2) (*) (MER-FS) (assembled) | Environmental; Aquatic; Non-marine Saline and Alkaline; Saline | (6) | 43.1 |
| Ga0306897_1000025 | 3300028373 | 36 884 | | Saline lake microbial communities from Rauer Islands, Antarctica - Metagenome Filla 1 #563 (v2) (*) (MER-FS) (assembled) | Environmental; Aquatic; Non-marine Saline and Alkaline; Saline | (6) | 43.3 |
| Ga0306871_1000094 | 3300028370 | 37 584 | | Saline lake microbial communities from Rauer Islands, Antarctica - Metagenome Hop E1 #497 | Environmental; Aquatic; Non-marine Saline and Alkaline; Saline | (5) | 43.6 |

| | | | | | | | |
|---|---|---|---|---|---|---|---|
| | | | | (v2) (*) (MER-FS) (assembled) | | | |
| Ga0306896_1000026 | 3300028369 | 36 884 | | Saline lake microbial communities from Rauer Islands, Antarctica - Metagenome Filla 1 #562 (v2) (*) (MER-FS) (assembled) | Environmental; Aquatic; Non-marine Saline and Alkaline; Saline | (6) | 43.9 |
| Ga0306883_100261 | 3300028365 | 22 119 | Identical to a part of Ga0306896_1000026 | Saline lake microbial communities from Deep lake, Antarctica - Metagenome #83 (v2) (*) (MER-FS) (assembled) | Environmental; Aquatic; Non-marine Saline and Alkaline; Saline | (6) | 35.4 |
| Ga0306898_1000102 | 3300028364 | 36 818 | | Saline lake microbial communities from Rauer Islands, Antarctica - Metagenome Filla 1 #564 (v2) (*) (MER-FS) (assembled) | Environmental; Aquatic; Non-marine Saline and Alkaline; Saline | (6) | 43.6 |
| Ga0306877_1000101 | 3300028363 | 37 488 | | Saline lake microbial communities from Deep lake, Antarctica - Metagenome #2 (v2) (*) (MER-FS) (assembled) | Environmental; Aquatic; Non-marine Saline and Alkaline; Saline | (6) | 43.6 |
| Ga0306872_100023 | 3300028361 | 71 274 | | Saline lake microbial communities from Rauer Islands, Antarctica - Metagenome Hop E1 #498 (v2) (*) (MER-FS) (assembled) | Environmental; Aquatic; Non-marine Saline and Alkaline; Saline | (5) | 34.7 |
| Ga0136586_1000173 | 3300012272 | 16 861 | | Saline lake microbial communities from Rauer Islands, Antarctica - Metagenome Filla 1 #562 (*) (MER-FS) (assembled) | Environmental; Aquatic; Non-marine Saline and Alkaline; Saline | (6) | 28.9 |
| Ga0136564_1000950 | 3300012263 | 8 312 | Identical to a part of Ga0136586_1000173 | Saline lake microbial communities from Club lake, Antarctica - | Environmental; Aquatic; Non-marine Saline | (6) | 15.9 |

| | | | | | | | |
|---|---|---|---|---|---|---|---|
| | | | | Metagenome #318 (*) (MER-FS) (assembled) | and Alkaline; Saline | | |

a. References:


1.Panwar P, Allen MA, Williams TJ, Hancock AM, Brazendale S, Bevington J, et al. Influence of the polar light cycle on seasonal dynamics of an Antarctic lake microbial community. Microbiome. 2020;8(1):1-24.

2.Hawley ER, Malfatti SA, Pagani I, Huntemann M, Chen A, Foster B, et al. Metagenomes from two microbial consortia associated with Santa Barbara seep oil. Marine genomics. 2014;18:97-9.

3.Hawley ER, Piao H, Scott NM, Malfatti S, Pagani I, Huntemann M, et al. Metagenomic analysis of microbial consortium from natural crude oil that seeps into the marine ecosystem offshore Southern California. Standards in Genomic Sciences. 2014;9(3):1259-74.

4.Sun M, Zhan Y, Marsan D, Páez-Espino D, Cai L, Chen F. Uncultivated Viral Populations Dominate Estuarine Viromes on the Spatiotemporal Scale. Msystems. 2021;6(2):e01020-20.

5.Williams TJ, Allen MA, Ivanova N, Huntemann M, Haque S, Hancock AM, et al. Genome Analysis of a Verrucomicrobial Endosymbiont With a Tiny Genome Discovered in an Antarctic Lake. Frontiers in microbiology. 2021;12.

6.Tschitschko B, Erdmann S, DeMaere MZ, Roux S, Panwar P, Allen MA, et al. Genomic variation and biogeography of Antarctic haloarchaea. Microbiome. 2018;6(1):1-16.
