# Supplementary material for: Virus-Host Interactions and Genetic Diversity of Antarctic Sea Ice Bacteriophages
Source: mBio. 2022 May 9;13(3):e00651-22. doi: 10.1128/mbio.00651-22 (PMC9239159; doi:10.1128/mbio.00651-22)
Supplement: FIG S4 [file mbio.00651-22-s0009.pdf]

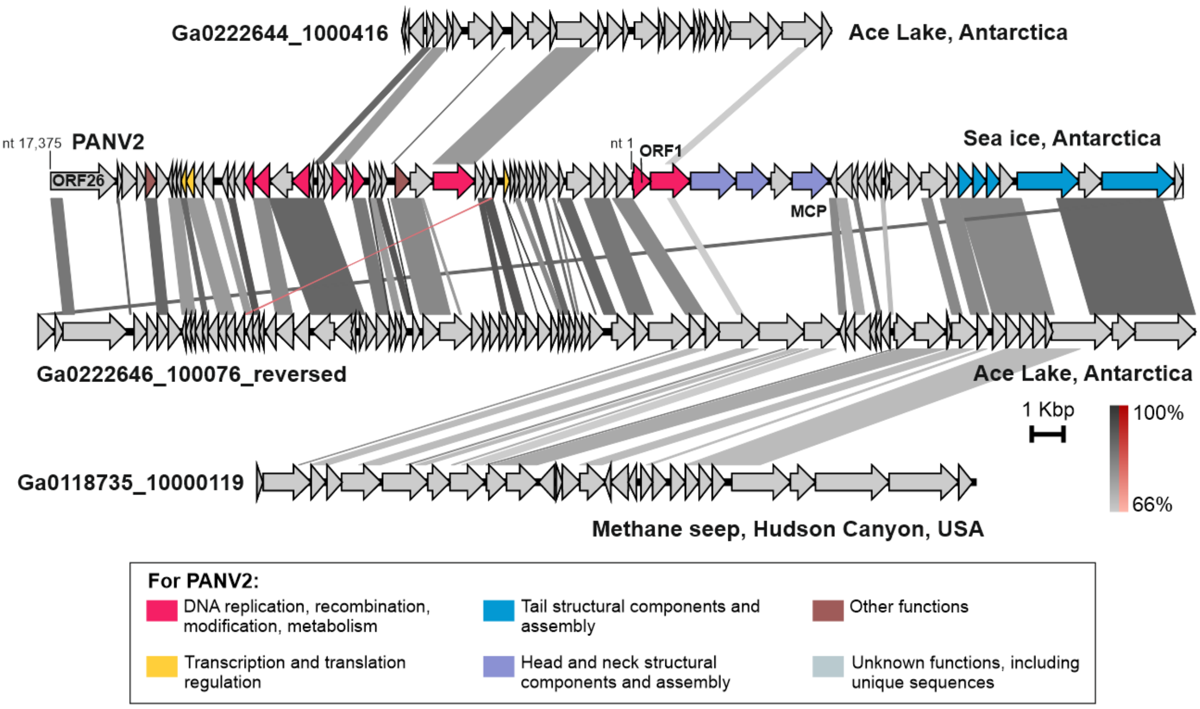


**Figure S4.** PANV2 and the selection of similar scaffolds found with blastn search against IMG/VR database. Full list of scaffolds is presented in Table S5. Here, those scaffolds that were identical to a part of some other scaffold are excluded. ORFs and genes are shown as arrows, and regions that are similar between sequences are shown as shadings (blastn, E-value threshold of 0.001, grey for direct and red for inverted similarities, from 66 to 100 %). Note that PANV2 genome is shown rearranged starting with nucleotide 17,375. Color codes for PANV2 ORFs are shown in the lower panel. Sampling locations are marked on the right. The figure was generated using Easyfig v. 2.2.2.
