# Supplementary material for: Virus-Host Interactions and Genetic Diversity of Antarctic Sea Ice Bacteriophages
Source: mBio. 2022 May 9;13(3):e00651-22. doi: 10.1128/mbio.00651-22 (PMC9239159; doi:10.1128/mbio.00651-22)
Supplement: FIG S5 [file mbio.00651-22-s0010.pdf]

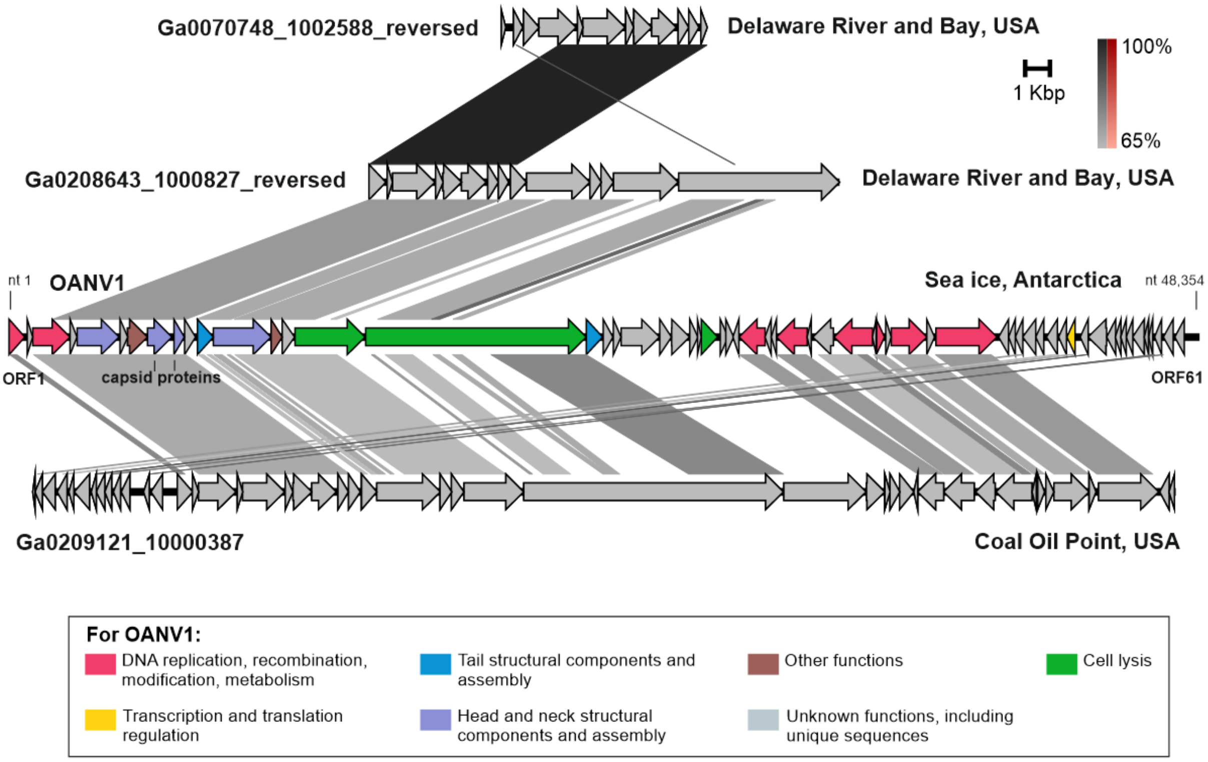

Figure S5. OANV1 and the selection of similar scaffolds found with blastn search against IMG/VR database. Full list of scaffolds is presented in Table S5. Here, those scaffolds that were identical to a part of some other scaffold are excluded. ORFs and genes are shown as arrows, and regions that are similar between sequences are shown as shadings (blastn, E-value threshold of 0.001, grey for direct and red for inverted similarities, from 65 to 100 %). Color codes for OANV1 ORFs are shown in the lower panel. Sampling locations are marked on the right. The figure was generated using Easyfig v. 2.2.2.
